# Self-organized tissue mechanics underlie embryonic regulation

**DOI:** 10.1038/s41586-024-07934-8

**Published:** 2024-09-11

**Authors:** Paolo Caldarelli, Alexander Chamolly, Aurélien Villedieu, Olinda Alegria-Prévot, Carole Phan, Jerome Gros, Francis Corson

**Affiliations:** 1 Developmental and Stem Cell Biology Department, Institut Pasteur, Université de Paris, CNRS UMR3738, Paris, France; 2 Collège Doctoral, Sorbonne Université, Paris, France; 3 Laboratoire de Physique de l’Ecole Normale Supérieure, CNRS, ENS, Université PSL, Sorbonne Université, Université de Paris, Paris, France; 4 Center for Studies in Physics and Biology, The Rockefeller University, New York, NY USA

**Keywords:** Biophysics, Gastrulation, Embryonic induction, Embryonic induction

## Abstract

Early amniote development is highly self-organized, capable of adapting to interference through local and long-range cell–cell interactions. This process, called embryonic regulation^1^, has been well illustrated in experiments on avian embryos, in which subdividing the epiblast disk into different parts not only redirects cell fates to eventually form a complete and well-proportioned embryo at its original location, but also leads to the self-organization of additional, fully formed embryos^2,3^ in the other separated parts. The cellular interactions underlying embryonic self-organization are widely believed to be mediated by molecular signals, yet the identity of such signals is unclear. Here, by analysing intact and mechanically perturbed quail embryos, we show that the mechanical forces that drive embryogenesis self-organize, with contractility locally self-activating and the ensuing tension acting as a long-range inhibitor. This mechanical feedback governs the persistent pattern of tissue flows that shape the embryo^4–6^ and also steers the concomitant emergence of embryonic territories by modulating gene expression, ensuring the formation of a single embryo under normal conditions, yet allowing the emergence of multiple, well-proportioned embryos after perturbations. Thus, mechanical forces act at the core of embryonic self-organization, shaping both tissues and gene expression to robustly yet plastically canalize early development.

## Abstract (web summary)

Mechanical forces act at the core of bird embryonic self-organization, shaping both tissues and gene expression to robustly yet plastically canalize early development.

## Main

**Fig. 1:**
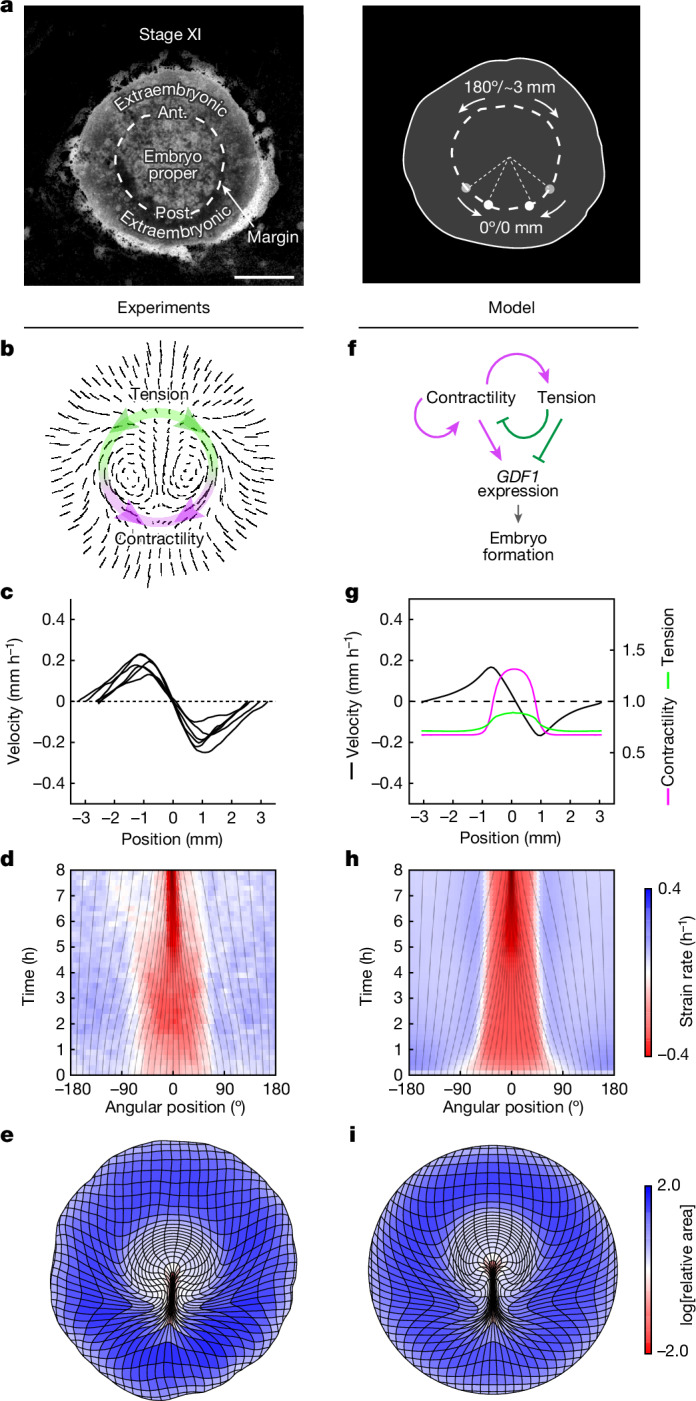
A model for self-organized force generation at the embryo margin. **a**, Transmitted light picture of a stage XI quail embryo, depicting embryonic territories (left) and the position in millimetres and degrees along the margin used to quantify tissue motion (arrows, right). Ant., anterior; post. posterior. **b**, Trajectories depicting the tissue flows obtained by particle image velocimetry (PIV) analysis at *t* = 6–8 h after the onset of tissue motion. The overlay denotes active contractility (magenta, highest in the posterior) and the resulting tension (green, approximately uniform along the margin) in the anterior margin. **c**, Profile of velocity along the margin from *n* = 6 biologically independent embryos from a previous study^6^ at *t* = 4 h (0 mm is posterior; around ±3 mm is anterior). **d**, The time evolution of strain rates along the margin. The grey lines denote the evolution of angular positions. 0° is posterior, ±180° is anterior. **e**, Deformation of an initially square grid from an average of six embryos^6^. **f**, The interplay between active contractility (magenta) and passive tension (green) proposed to regulate embryo formation through *GDF1* expression. The solid arrows indicate local feedback arising from the response to tissue contraction versus stretching (see Extended Data Fig. 1). The dashed arrow indicates long-range tension propagation. **g**, Predicted profiles of contractility (magenta), tension (green) and velocity (black) at *t* = 4 h. **h**, Time evolution of strain rates along the margin. **i**, Global tissue deformation when self-organizing contractility is implemented in a in 2D fluid-mechanical model. Colours in **d**, **h**, **e** and **i** quantify contraction (red) and expansion (blue). Scale bar, 1 mm (**a**).

Following Turing’s seminal work on spontaneous pattern formation by diffusing chemicals^7^ and the advent of molecular biology, studies of embryonic regulation have mostly focused on secreted factors that could mediate the underlying cell–cell interactions. The redirection of cell fates to form ectopic embryos in chicken epiblast subdivision experiments has been shown to involve GDF1 (also known as cVg1)—a TGF-β-superfamily secreted molecule that is normally restricted to the posterior side of the margin between the embryonic and extraembryonic territories (Fig. 1a); ectopic expression of *GDF1* at other locations along the margin accompanies the emergence of additional embryos in separated epiblast parts^8–10^. As GDF1 is both necessary and sufficient to trigger embryo formation, a long-range, fast-diffusing inhibitor of *GDF1* expression emitted from the posterior has been postulated to explain the formation of a single embryo in intact epiblasts and the emergence of ectopic embryos after subdivision, as the separated parts are freed from inhibition^11,12^. However, in a tissue that is millimetres across, it is unclear that interactions mediated by a diffusing inhibitor could support the rapid redirection of *GDF1* expression, downstream of the transcription factor PITX2, which is detected as early as 3 h after epiblast separation^13^ (the time needed for transport by diffusion grows as the square of distance; an estimate of around 2 h for a small protein to diffuse over 1 mm (ref. ^14^) yields around 8 h for the approximately 2 mm between the posterior and anterior ends of the margin). To date, a candidate for this role remains to be identified. Thus, although a number of molecular players have been identified, how the embryo regulates and, by implication, how the embryo self-organizes, remains unclear. On the other hand, diffusing molecules are just one way in which cells can communicate. Turing himself recognized that the mechanical state of an embryo could be equally relevant, and theoretical models have since been proposed to explore its role in self-organized processes such as the patterning of feather primordia, the limb skeleton^15–17^ or polarized cell shapes in embryonic epithelia^18^. In the context of embryonic regulation, the reallocation of cell fates must be accompanied by a redirection of morphogenesis, but whether and how molecular and mechanical cues combine has received little attention.

We have recently shown that a supracellular actomyosin ring assembles at the embryo margin, and that its graded contractile activity (decaying from posterior to anterior) powers the large-scale rotational tissue motion that shapes the early embryo^4–6^ (Fig. 1a–c and Supplementary Video 1). Thus, the entire margin is not only a molecular but also a mechanical organizer of development, whereby the shape and pattern of the embryo are simultaneously actualized. Here, using quantitative image analysis, mathematical modelling and mechanical perturbation experiments, we investigated whether passive tissue tension, which is induced throughout the margin by active contraction in its posterior and rapidly released after cutting, could act as a fast-propagating and long-range inhibitor impinging on gene expression to control embryonic self-organization.

## Modelling self-organized force generation

As the early embryo is shaped by active forces generated along its margin, we anticipated that redirection of embryogenesis in response to perturbations should require a redirection of force generation at the margin. In the intact epiblast, an indication of the regulation of force generation came from the kinematics of tissue motion along the margin, as observed in quail embryos expressing a membrane-bound green fluorescent protein (memGFP)^6^. Profiles of velocity and the time evolution of strain rates along the embryo margin revealed a pattern of two domains of relatively uniform contraction in the posterior and stretching in the anterior (notice the triangular velocity profiles in Fig. 1c, with upward and downward slopes corresponding to stretching and contraction, respectively), which maintained stable proportions over time, even though the tissue continuously converges towards the posterior (Fig. 1d). The persistence of these domains over hours of development, even as critical regulators (*GDF1*, but also *NODAL*, *FGF8*, and *WNT* and planar cell polarity genes) are advected into the emergent streak^10,19–21^, hinted to a continuous regulation and a degree of autonomy of mechanics from molecular signalling. Having previously observed that the transient supracellular actomyosin cables that make up the margin exhibit opposite dynamics in these two domains—steadily contracting in the posterior, extending and breaking up in the anterior^6^—we reasoned that the modulation of their collective contractile activity (hereafter, contractility) in response to different tissue-deformation rates could underlie the maintenance of a stable pattern of forces at the margin.

**Extended Data Fig. 1:**
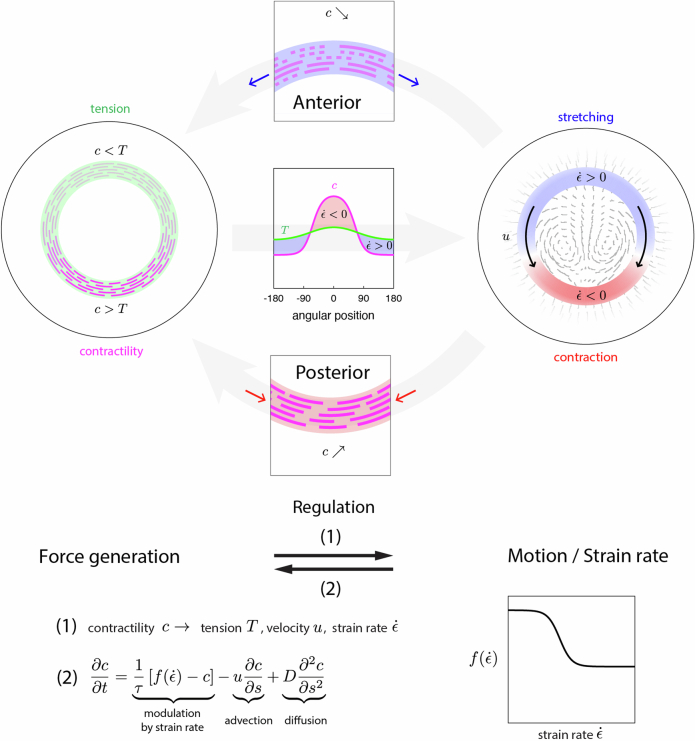
Mechanical feedback underlying self-organization of force generation and tissue flows. Contractile actomyosin cables (magenta segments in left panel) generate tension that propagates along the margin (green). An imbalance in contractility (lighter magenta segments in the anterior denote lower contractility, from lower cable density or activity) and therefore tension drives tissue flows across the embryonic disk (right panel) and differential contraction of the margin (red: contraction; blue: stretching). As depicted in the central plot, in the posterior contractility dominates over tension and the margin contracts, whereas in the anterior tension propagating from the posterior dominates over contractility and the margin extends. This is symbolized by equation (1), which takes different forms in the 1D linear and 2D nonlinear models (see Methods and Supplementary Discussion). Feedback occurs through the modulation of margin contractility by the strain rate, whereby stretching inhibits contractility, as schematized by the boxed panels (dotted lines depict cable breakdown under stretching) and described by equation (2); the transition from high to low contractility under increasing strain rate is described by the sigmoidal function $f$, plotted to the right of the equations.

To examine this possibility, we formulated a minimal one-dimensional (1D) model for the regulation of force generation at the margin, represented as a tensile line in which contractility is upregulated in regions undergoing contraction and downregulated in regions undergoing extension (Fig. 1f, Methods and Extended Data Fig. 1). With the local rate of contraction or extension depending on the balance between active contractility and the passive tension induced by contractility elsewhere along the margin, contractility locally self-activates, whereas the tension that it induces acts as a long-range inhibitor. The model also incorporates a diffusion term, representing the spreading of contractility as new cell–cell junctions are recruited into cables, as previously observed in avian gastrulation^22^ and also reported during the propagation of a runaway contraction wave in the *Drosophila* embryo^23^ (a graphic representation of the model’s components is shown in Extended Data Fig. 1). Similar to a previously proposed model for cell polarization in epithelia^18^, this is in effect a mechanical analogue of a Turing reaction–diffusion model^7^ and, like a Turing model, it can support the spontaneous emergence and stable maintenance of domains of high and low contractility, correlating with differential behaviours of the supracellular force-generating machinery.

To describe the full two-dimensional (2D) pattern of tissue motion during embryogenesis, self-organized margin contractility, as described above, was incorporated into a fluid-mechanical model of tissue flows in the epiblast^6^, with an initial bias in contractility representing the pre-existing polarity that directs embryo formation at the posterior margin^10^. The model also allows for a nonlinear, saturating relationship between the tension borne by contracting actomyosin cables and the rate at which they contract (as in ref. ^24^), a refinement that we later show is required to account for the response to cutting (the model would otherwise predict a jump in the contraction rate of posterior halves after the release of margin tension). The resulting model recapitulates the profiles of tissue velocity along the margin (Fig. 1g (black curve)), the maintenance of actively contracting and passively stretched domains with stable proportions (Fig. 1h) and the pattern of tissue motion entrained by the margin across the disk. Whereas our minimal 1D model is, mathematically at least, very similar to a molecular Turing model, this extended model identifies several effects specific to mechanics that have a critical role in the emergence of a single embryo. At odds with our minimal 1D model, in which tension propagates unopposed, in 2D, its range is restricted by the ‘drag force’ from the surrounding tissue, and force transmission along the margin must dominate over propagation to the surrounding tissue to support inhibition in the anterior margin. Mechanical regulation must also be fast enough to counteract advection towards the posterior and maintain a stable contractile domain.

With parameter values chosen to satisfy these conditions (Supplementary Methods B), self-organized contractility at the margin, implemented in a 2D fluid-mechanical model of gastrulation, recapitulates the full pattern of tissue motion that leads up to the formation of the primitive streak, the hallmark of the primary embryonic axis (compare Fig. 1e and 1i; Supplementary Video 1).

## Tissue contractility regulates *GDF1*

**Fig. 2:**
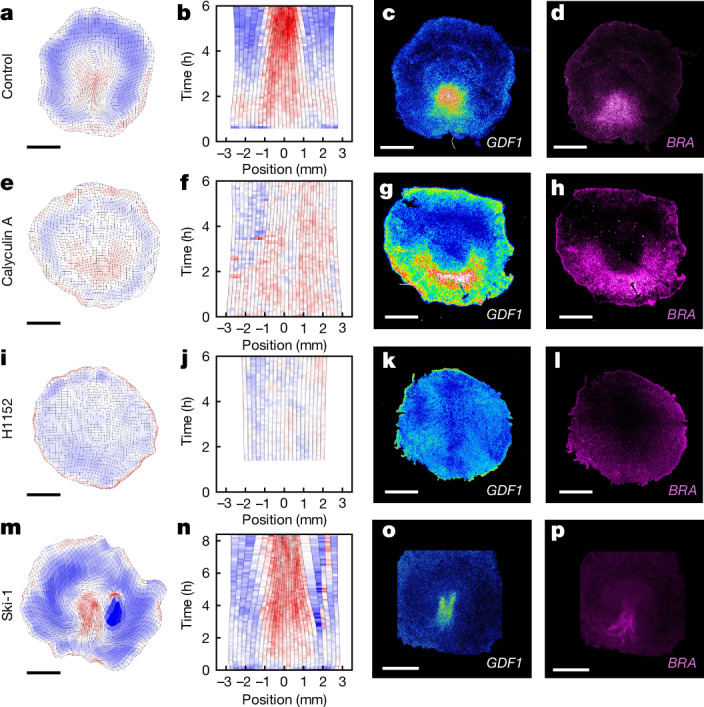
Tissue contractility modulates *GDF1* expression in intact epiblasts. **a**–**f**, Tissue deformation maps (**a**,**e**,**i**,**m**), time evolution of strain rates along the margin (**b**,**f**,**j**,**n**; 0 mm is posterior) and corresponding expression of *GDF1* (**c**,**g**,**k**,**o**) and *BRA* (**d**,**h**,**l**,**p**) in control (**a**–**d**; *n* = 32), calyculin-A-treated (**e**–**h**; *n* = 18), H1152-treated (**i**–**l**; *n* = 13) and Ski-1-treated (**m**–**p**; *n* = 6) biologically independent embryos. Colours in **a**, **b**, **e**, **f**, **i**, **j**, **m** and **n** quantify contraction (red) and expansion (blue). Scale bars, 1 mm (**a**, **c**, **d**, **e**, **g**, **h**, **i**, **k**, **l**, **m**, **o** and **p**).

**Extended Data Fig. 2:**
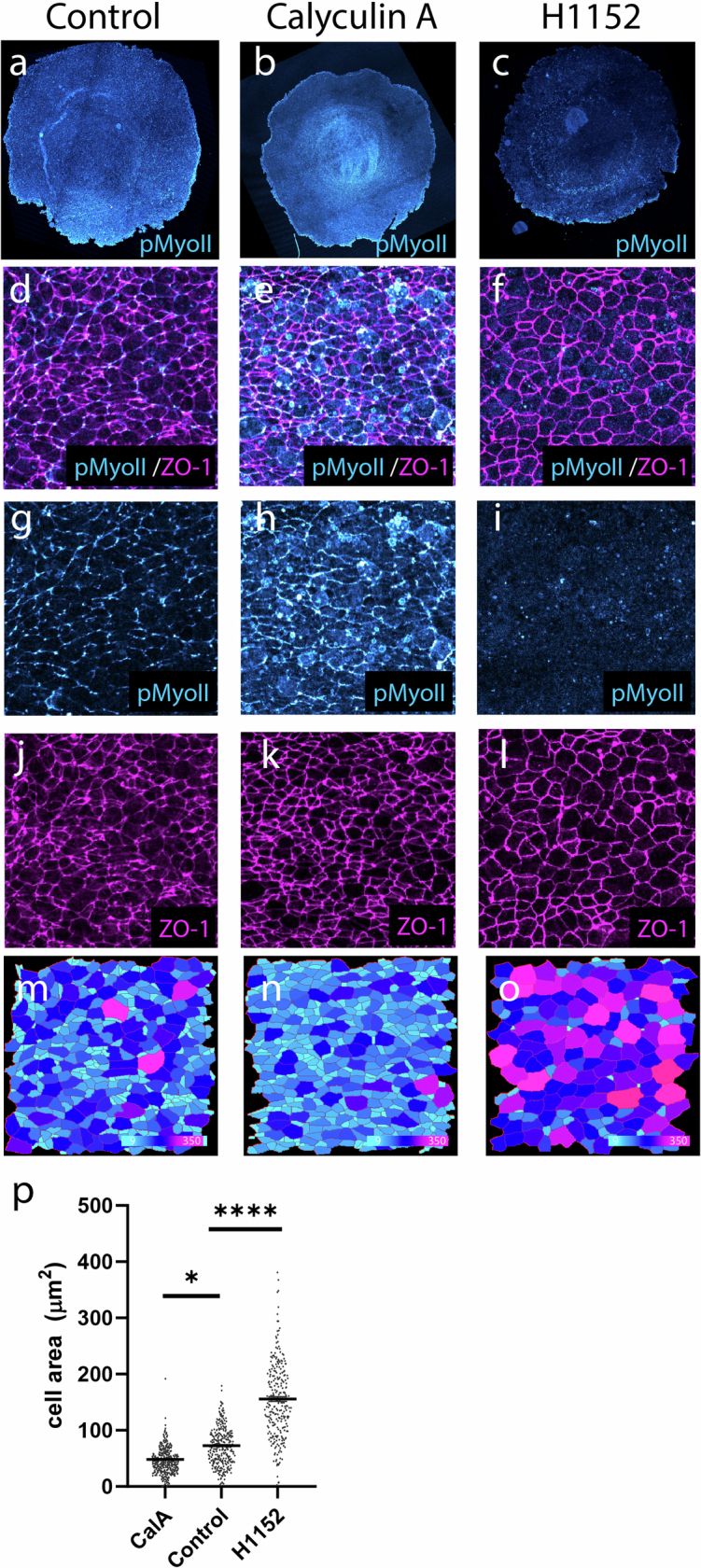
Effect of Calyculin A and H1152 on Myosin II phosphorylation and apical cell area. **(a-c)** Control (a), Calyculin A- (b) and H1152-treated (c) embryos immunostained using anti-pMyosin II antibodies (cyan). **(d-l)** Higher magnification in the posterior margin region showing pMyosin II (cyan) and ZO-1 (magenta) staining in control (d, g, j), Calyculin A- (e, h, k), and H1152- (f, i, l) treated embryos. **(m-o)** Colour-coded apical area of cells segmented using the ZO-1 staining. **(p)** Quantification of cell area; Calyculin A: n = 4 biologically independent embryos, 1914 cells; Control: n = 4 biologically independent embryos, 1059 cells; H1152: n = 3 biologically independent embryos, 629 cells; Mean ± s.e.m; *p = 0,0193, ****p < 0.0001; Nested one-way ANOVA. Source Data

**Extended Data Fig. 3:**
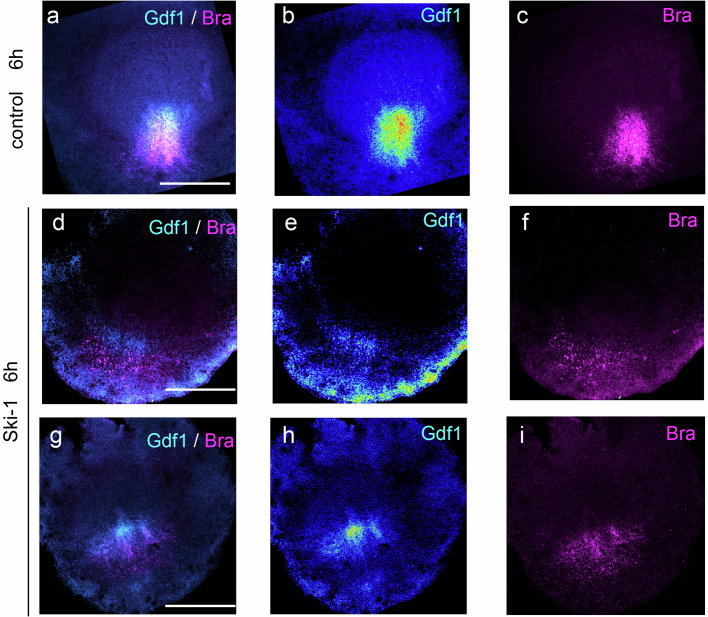
Ski-1 treatment interferes with Gdf1 and Bra expression. **(a-i)** Control (a-c, n = 10 biologically independent embryos) and Ski-1-treated embryos (d-i, n = 17 biologically independent embryos) showing overall decreased (d-f) and irregular (g-i) expression of Gdf1 (cyan/rainbow LUT) and Bra (magenta). Scale bar is 1 mm.

In our model, the balance between active contractility and passive tension along the margin governs the formation of a single contracting primitive streak. Given the above-mentioned critical role of *GDF1* expression in primitive streak (and embryo) formation, to be effective, such a mechanical regulation must impinge on *GDF1* expression, as shown in Fig. 1f. We therefore tested whether the mechanical state of the margin (contracting versus stretching), which is itself controlled by the difference between contractility and tension, acts as a regulator of *GDF1* expression, ensuring its posterior restriction in the intact epiblast. To manipulate tissue contractility, we incubated embryos in the presence of calyculin A and H1152 inhibitors, which increase and decrease myosin activity, respectively. In calyculin-A-treated and H1152-treated embryos, we observed increased and decreased levels of phosphorylated (that is, active) myosin, respectively, as well as reduced and increased apical cell areas at the margin, respectively, compared with the control embryos (Extended Data Fig. 2), confirming that these drugs act on myosin activity and cell contractility. Notably, although calyculin A and H1152 have opposite effects on cell contractility, they both impaired the overall tissue flows (Fig. 2e,f,i,j), as expected if differences in contractility along the margin are required to drive net tissue flow^6^. In H1152-treated embryos, the margin did not contract and, instead, expanded over time (Fig. 2j); by contrast, in calyculin-A-treated embryos, motion appeared to stall as the result of a more-even contraction, with the total length of the margin decreasing as in the controls (Fig. 2f). On the other hand, increasing and decreasing tissue contractility had opposite effects on the expression of *GDF1* and brachyury (*BRA*), its downstream target and marker of the primitive streak. After only 4–5 h of incubation with H1152 or calyculin A, we found that *GDF1* and *BRA* expression was abolished and expanded, respectively, at the margin (Fig. 2g,h,k,l), in congruence with the mechanical state at the margin (which, in H1152-treated embryos, compounds the extension of the margin as a whole and areal expansion of individual cells). We next investigated the molecular mechanism through which contractility regulates expression. Mesoderm induction has been shown to be controlled by the evolutionarily conserved β-catenin mechanosensitive pathway in *Nematostella*^25^, *Drosophila*^26^, zebrafish^27^, as well as in patterned colonies of human embryonic stem cells modelling human gastrulation^28^. In avians, the critical function of *GDF1* in primitive-streak formation has been shown to require the synergistic activity of the β-catenin pathway, which was attributed to the activity of WNT^29^. We therefore tested whether β-catenin activity during avian gastrulation is also mechanosensitive and controls *GDF1* expression. To do so, we incubated embryos with Ski-1, a SRC kinase inhibitor that, in the context of the avian skin^30^, has been shown to efficiently prevent the mechanosensitive phosphorylation of β-catenin, which itself enables its translocation from the membrane to the nucleus. In Ski-1-treated embryos, tissue flows proceeded normally, with cells converging to shape the incipient primitive streak. However, gene expression analysis revealed that *GDF1* and its target *BRA* were overall downregulated, and exhibited an irregular expression, occasionally resembling twinned primitive streaks within the posterior contracting domain (Fig. 2 and Extended Data Fig. 3), indicating that *GDF1* expression is regulated at least in part by the mechanosensitive β-catenin pathway.

Taken together, these results show that tissue contractility (through the mechanical state of the tissue) regulates *GDF1* and *BRA* expression at the margin, and suggest that this regulation involves the SRC-dependent mechanosensitive β-catenin pathway. They further support the view that self-organized tissue mechanics control the formation of a single embryo in the unperturbed epiblast (Fig. 1f).

## Mechanics drive ectopic embryo formation

**Fig. 3:**
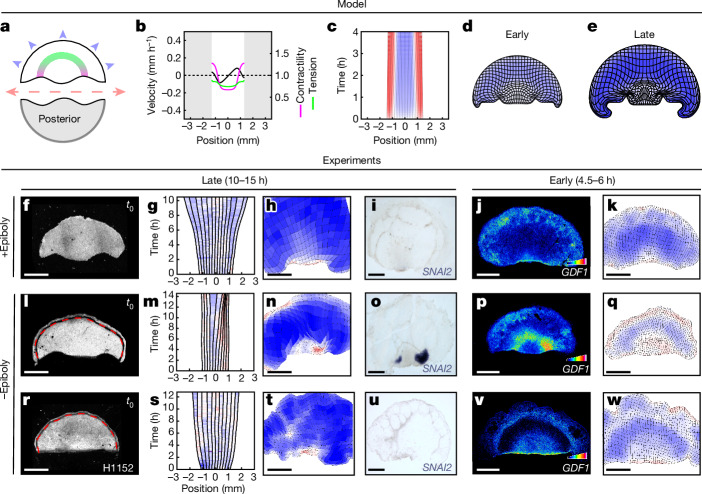
Self-organized tissue mechanics drives ectopic embryo formation after epiblast subdivision. **a**–**e**, Model predictions for the response to epiblast bisection in anterior halves. **a**, Sketch of epiblast bisection. **b**–**e**, Predicted contractility (magenta), tension (green) and velocity (black) profiles at *t*_0_ + 4 h (**b**), kymograph of margin strain rate (**c**; 0 mm is anterior) and deformation maps at 4 h (**d**) and 8 h (**e**) after bisection. **f–w**, UV-laser dissected anterior epiblast halves with (**f**–**k**) or without (**l**–**w**) epiboly and treated with H1152 (**r**–**w**). The red dashed lines indicate the UV cut abrogating the epiboly process. **f**,**l**,**r**, A memGFP embryo at *t*_0_. **g**,**m**,**s**, Kymographs of the strain rates along the margin; 0 mm is anterior. **h**,**n**,**t**, Deformation maps at 10 h (**h**), 15 h (**n**) and 13 h (**t**) after epiblast bisection. **i**,**o**,**u**, *SNAI2* expression in the same embryos fixed after live imaging (*n* = 8 out of 8 biologically independent embryos with epiboly; *n* = 5 out of 5 without epiboly (3 out of 5 show two ectopic primitive streaks, 2 out of 5 show one ectopic primitive streak); *n* = 4 out of 4 without epiboly and with H1152). **j**,**k**,**p**,**q**,**v**,**w**, *GDF1* expression and the corresponding deformation maps at 4.5 h (**j**,**k**,**p**,**q**) and 6 h (**v**,**w**) after epiblast bisection. Scale bars, 1 mm (**f**, **h**–**k**, **l**, **n**–**q**, **r** and **t**–**w**).

The classic epiblast subdivision experiments that revealed the regulative nature of early avian embryos have been so far interpreted in molecular terms (see the above-mentioned diffusing inhibitor model for the regulation of *GDF1* expression). However, cutting is a mechanical perturbation, the most immediate consequence of which is to interrupt the propagation of tension along the embryo margin^6^. Our model predicts that—in the absence of propagated tension, which normally acts as negative regulator—ectopic contraction foci spontaneously emerge. Assuming an initial posterior bias in the gradient of contractility at the margin, as required to direct embryo formation in the posterior in the intact epiblast, two self-sustained contraction foci (and two streaks) are predicted to emerge at the posterior-most positions along the margin (Fig. 3a–e and Supplementary Video 3).

**Extended Data Fig. 4:**
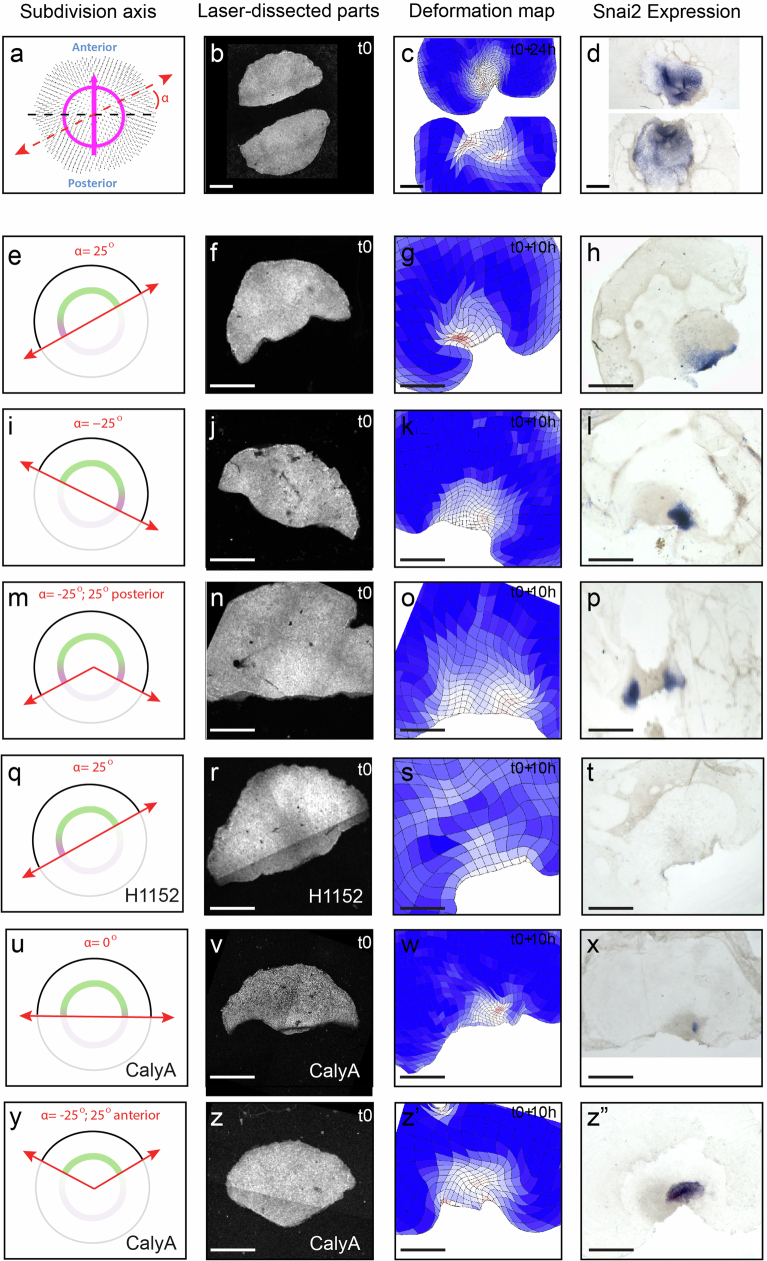
Formation of ectopic embryos in anterior parts bisected at varying angles. **(a-d)** Experimental procedure for the evaluation of bisection angles on ectopic embryo formation in anterior parts. Epiblasts are bisected with an angle that is defined following the automated detection of the margin and anteroposterior axis using PIV analysis [(a); velocity fields, black arrows; margin and anteroposterior, in magenta; position of cut defined by the angle α, dotted red lines)]. Anterior epiblast parts are bisected using a UV-laser (b). The formation of ectopic axes is monitored by tissue deformation (c) and verified by (d) expression of Snai2. **(e-x)** Bisection experiments for different values of α (e, i, m, q, u, y) showing anterior epiblast parts at t0 (f, j, n, r, v, z), the deformation maps after 10 h (g, k, o, s, w, z’) and the corresponding Snai2 expression (d, h, l, p, t, x, z”). Scale bars, 1 mm. (e, n = 3/3; i, n = 6/6; m, n = 2/2; q, n = 2/2; u,y n = 5/5 biologically independent subdivided epiblasts).

To test a role for self-organized contractility in the redirection of *GDF1* expression and the formation of ectopic embryos in anterior halves, we revisited classic epiblast bisection experiments^3^, with the added power of our quantitative analysis tools. Intact epiblasts were analysed using live imaging for 1–2 h, and tissue motion was analysed in real time to locate the margin and presumptive axis (Methods), allowing a precise bisection into anterior and posterior halves using ultraviolet (UV) laser dissection. The anterior halves were then cultured for 4.5–6 h to check for expression of *GDF1*, or left to develop for 10–15 h and checked for *SNAI2*, a late primitive streak marker expressed like *BRA* at this stage (Extended Data Fig. 4a–d and Supplementary Video 4). Unexpectedly, we did not observe ectopic primitive streaks in the anterior halves in these conditions, based on either tissue deformation or *GDF1* and *SNAI2* expression (Fig. 3f–k and Supplementary Video 5). Ectopic primitive streaks only developed when epiblasts were bisected at an angle, such that the two halves had an overlap with the posterior domain of active contraction (Extended Data Fig. 4e–p). At odds with spontaneous symmetry breaking in the model, this suggested that the anterior margin may not support ectopic embryo formation in the absence of an activating trigger. However, analysing tissue deformation in anterior halves, we noted that the margin and the embryonic territory as a whole were abnormally stretched (Fig. 3g,h; tissue expansion is shown in blue) compared with in intact epiblasts (Fig. 1d) or biased anterior halves (Extended Data Fig. 4). This suggested that the tension imposed on the embryonic disk by epiboly (the process by which the epiblast spreads through the migration of its edges on the vitelline membrane) might oppose the emergence of contractile domains at the embryo margin. Indeed, when epiboly was abrogated by uncoupling the migrating edge from the rest of the epiblast using laser dissection (Fig. 3l (dotted red lines) and Supplementary Video 3), we observed the rapid emergence of contraction foci (within 1–2 h) that expressed *GDF1* (by 4.5 h) and eventually elongated into one or two *SNAI2*^+^ primitive streaks (Fig. 3m–q). Thus, the anterior margin does have the potential to initiate self-sustained contraction after bisection, followed by *GDF1* expression and streak formation. However, this potential is susceptible to the counteracting effect of epiboly-induced tissue tension.

To corroborate a role for margin contractility as an upstream regulator of *GDF1* and primitive streak emergence after bisection, we incubated bisected epiblasts with H1152 or calyculin A, delivered uniformly in the culture medium. Whereas H1152 treatment prevented margin contraction, *GDF1* expression and subsequent primitive streak formation in epiboly-abrogated anterior halves (Fig. 3r–w), as well as in biased anterior halves (Extended Data Fig. 4q–t), calyculin A treatment allowed margin contraction to overcome epiboly-induced stretching to form primitive streaks in anterior parts (Extended Data Fig. 4u–z″). Taken together, these results clarify the processes underlying classic subdivision experiments and demonstrate that, after physical cutting, redirection of tissue motion, through self-organized contractility, is the first event acting upstream of *GDF1* expression to drive primitive-streak formation in anterior halves.

## Mechanical feedback (re)scales territories

**Fig. 4:**
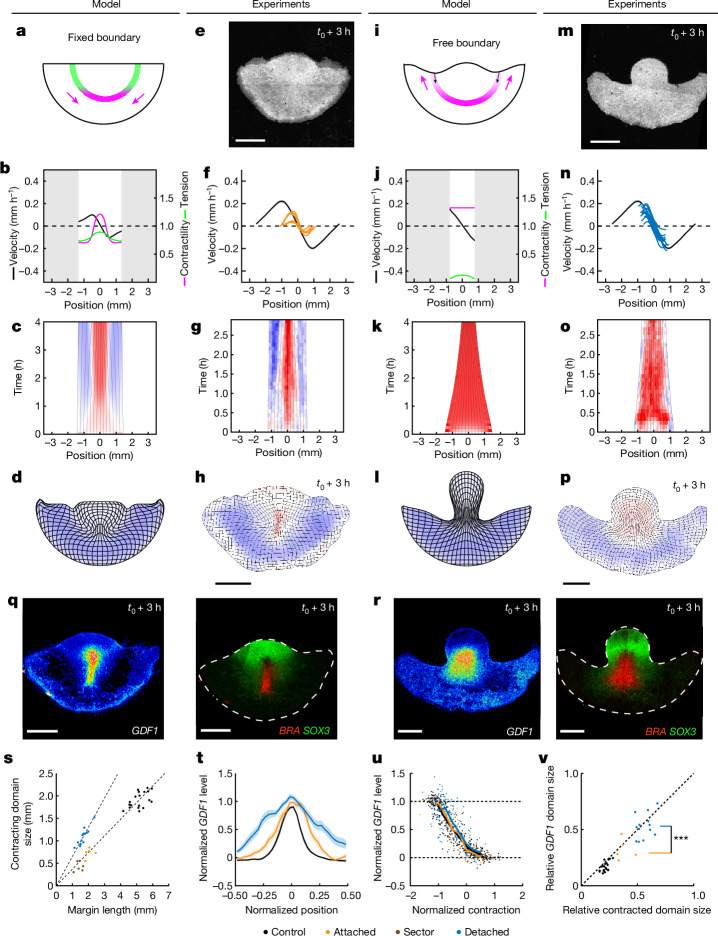
Mechanical feedback rescales embryonic territories. **a–p**, Model predictions (**a**–**d** and **i**–**l**) and experimental responses (**e**–**h** and **m**–**p**) to fixed and free boundary conditions in posterior epiblast halves. **a**,**e**,**i**,**m**, Sketches of the experiment (**a**,**i**) and memGFP posterior epiblast half after 3 h (**e**,**m**). **b**,**f**,**j**,**n**, Predicted and experimental profiles of contractility (magenta), tension (green) and velocity (in **f** and **n**, the black lines show the mean ± s.e.m. velocity profile from *n* = 19 control intact biologically independent embryos, shown in Extended Data Fig. 5b; the orange lines show *n* = 6 biologically independent embryos; and the blue lines show *n* = 13 biologically independent embryos) along the margin at *t*_0_ + 2 h. **c**,**g**,**k**,**o**, Kymographs of strain rates along the margin. **d**,**h**,**l**,**p**, Deformation maps at the end of the experiments and simulations (*t*_0_ + 3–4 h). **q**,**r**, *GDF1*, *BRA* and *SOX3* mRNA in the embryos shown in **e**–**h** and **m**–**p**, respectively, fixed after live imaging. **s**, The size of the contracting domain in controls (black; *n* = 19 biologically independent embryos), attached halves (orange; *n* = 6 biologically independent embryos), smaller attached sectors (brown; *n* = 6 biologically independent embryos; see Extended Data Fig. 6a–c) and detached halves (blue; *n* = 13 biologically independent embryos) versus total margin length. The dotted lines show slopes of 0.37 for the controls and attached halves/sectors (*R* = 0.97), respectively, and 0.65 for detached halves (*R* = 0.90). **t**, Intensity profiles of the *GDF1* mRNA signal along the margin in different conditions (colours are as described in **s**; see also Extended Data Fig. 5g for profiles of contraction, *BRA* and *SOX3*; the intensity profiles are normalized to the margin length). Data are mean ± s.e.m. **u**, *GDF1* level versus contraction (Methods) in portions of the margin (the solid lines show the average for each condition; colours are as described in **s**). **v**, The relative size of the *GDF1* domain versus the relative size of the contracted domain (Methods; *R* = 0.91; ***P = 0.0003, one-sided permutation test, *n* = 10^6^ random permutations; colours are as described in **s**). Colours in **c**, **d**, **g**, **h**, **k**, **l**, **o** and **p** quantify contraction (red) and expansion (blue) as in Fig. 1. Scale bars, 1 mm (**e**, **h**, **m**, **p**, **q** and **r**). Source Data

**Extended Data Fig. 5:**
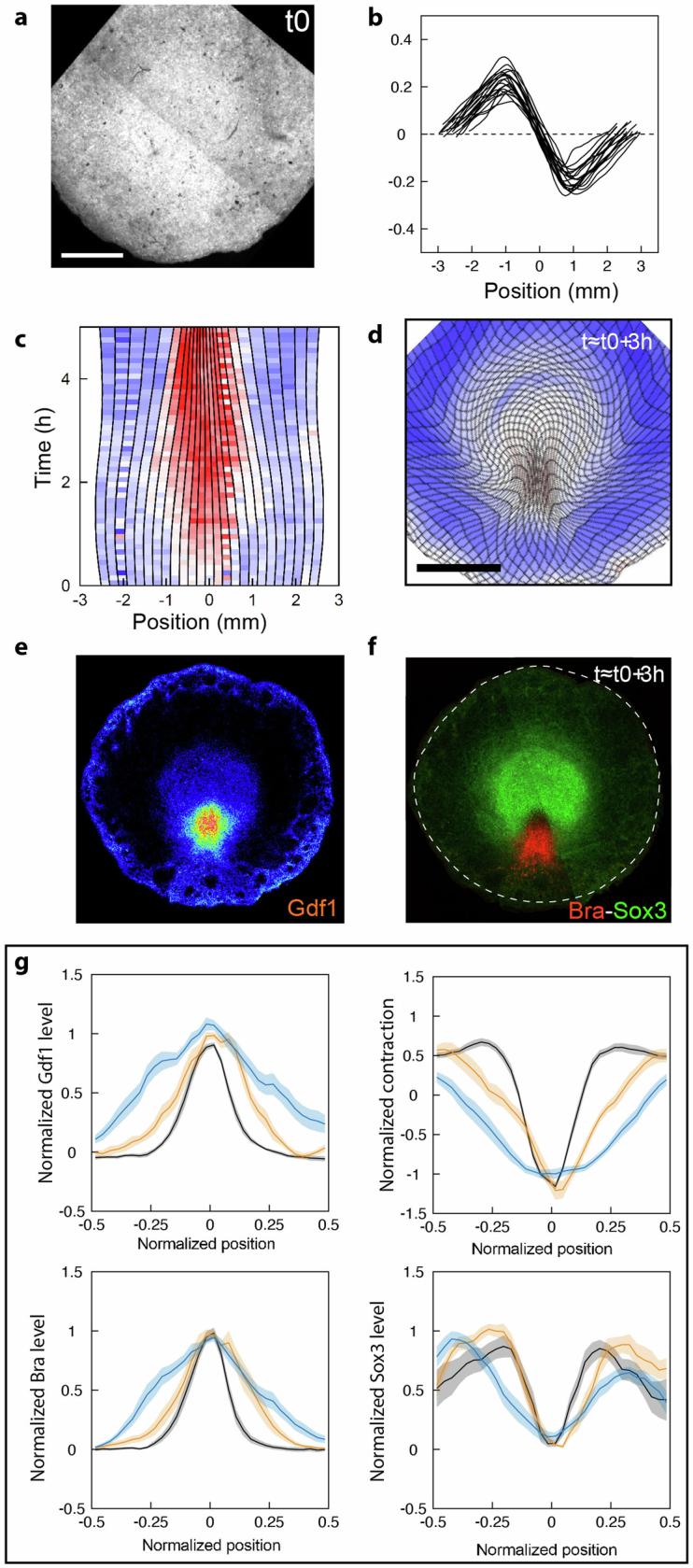
Tissue motion and gene expression in intact epiblasts and posterior halves. **(a-f)** Representative example of an intact epiblast whose developmental timing is precisely matched to posterior halves with free/attached boundary condition 3 h after bisection (t = 5 h ≈ t0 + 3 h, see Methods for details on the procedure). (a) Intact epiblast at t0. (b) Velocity profiles at the margin (n = 19; 0 mm is posterior). (c) Kymograph of strain rates at the margin, deformation map (d), and corresponding expression of Gdf1 (e), Brachyury and Sox3 (f) at t ≈ t0 + 3 h. **(g)** Mean ±SE profiles of normalized mRNA signal intensity and contraction at the margin in intact epiblasts (black, n = 19 biologically independent embryos) and posterior halves with attached (orange, n = 6 biologically independent embryos) and free (blue, n = 13 biologically independent embryos) edges. Scale bars, 1 mm.

**Extended Data Fig. 6:**
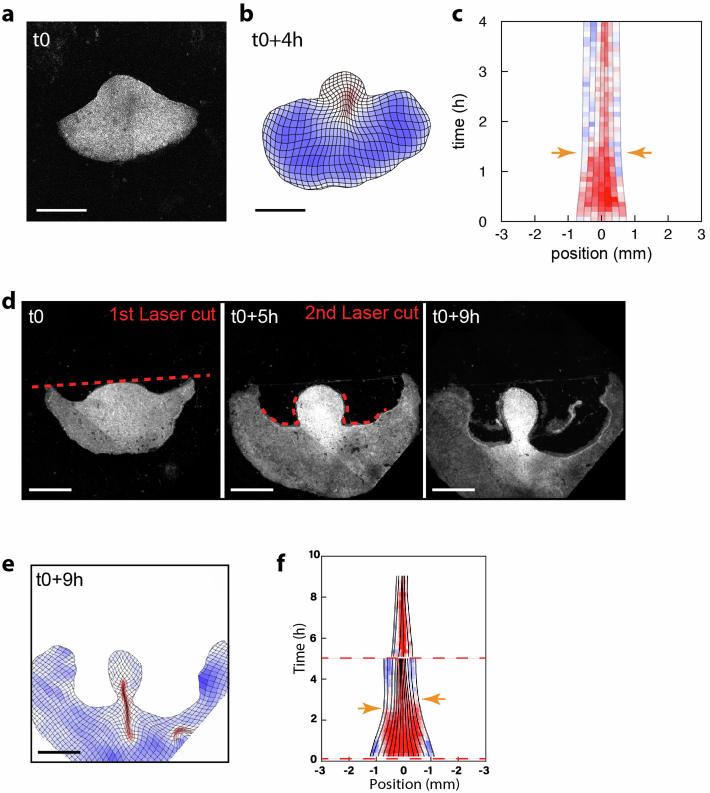
Development of posterior parts with different sizes and at long times. (**a**-**c**) Mechanical rescaling in a posterior sector <180° with reattached edges. Snapshot of a ~120° posterior sector following ablation (t0, a), deformation map at t0 + 4 h (b), and kymograph of strain rates along the margin (c; 0 mm is posterior; orange arrows indicate when edges reattach). (**d**-**f**) Long-term development of posterior half with repeated laser cuts to release border attachment. (d) Time series of a posterior epiblast half just after the 1^st^ laser cut bisecting the epiblast (t0, left panel), just before the 2^nd^ laser cut, as embryo borders reattach (t0 + 5 h, middle panel), and after the entire margin has converged and contributed to the forming primitive streak (t0 + 9 h, right panel). (b) Deformation map (t0 + 9 h). (f) Corresponding kymograph of strain rates at the margin (0 mm is posterior; orange arrows indicate when epiblast borders reattach; dotted red lines correspond to successive laser cuts). Scale bars, 1 mm.

While previous studies of embryonic regulation have given greatest attention to the emergence of ectopic embryos, our mechanical self-organization model also makes stringent predictions for the development of well-proportioned embryos from posterior halves. Indeed, the model predicts that the domains of active contraction and stretching adjust their proportions to tend towards a preferred homeostatic tension along the margin, shrinking if the tension is too large or expanding when it is too low. It naturally follows that these domains should scale with margin length to restore homeostasis, as demonstrated in simulations of posterior halves (Fig. 4a–d). However, critically, this can only occur if the boundary of the tissue is fixed (if the cut edge of the epiblast reattaches to the vitelline membrane). If instead the boundary of the tissue is free, the tension along the margin cannot build up again after it is released by cutting, and the actively contracting domain grows uninhibited to occupy the entire margin (Fig. 4i–l). By preparing posterior halves in different ways, we could favour or disfavour reattachment and challenge these predictions. As predicted, in epiblast halves that reattached, the contracting and stretching domains scaled down to occupy the same proportions of the half-length margin (Fig. 4e–g,s and Extended Data Fig. 5) so that the emerging primitive streak (the posterior region undergoing convergent extension) rescaled according to the new epiblast size (Fig. 4h and Supplementary Video 5). Similarly, in smaller portions of the epiblast (that is, a 120° posterior sector), the contracting domain rescaled to reach the same proportion of the shorter remaining margin, demonstrating the scaling behaviour predicted to emerge from self-organized tissue mechanics (Fig. 4s and Extended Data Fig. 6). By contrast, in epiblast halves that did not reattach, contraction spread to the entire margin and the primitive streak did not rescale (Fig. 4n–p,s). Notably, the margin did not contract faster in epiblast halves with free edges (Fig. 4n), implying that contraction is not limited by tension along the margin in the intact epiblast. This justifies our choice of a model in which the contraction rate of cellular junctions tends to a plateau when contractility dominates over tension.

Having demonstrated a rapid redirection of tissue motion after cutting that is controlled by mechanical boundary conditions, we examined whether this carries over to gene expression patterning. Indeed, just 3 h after cutting, we observed a narrowing of the *SOX3*^+^ (neuroectodermal) and the *BRA*^+^*GDF1*^+^ (mesendodermal) territories in epiblast halves with a reattached edge (Fig. 4q). By contrast, in epiblast halves with a free edge, the fraction occupied by the *BRA*^+^*GDF1*^+^ mesendodermal territory grew at the expense of the *SOX3*^+^ ectodermal territory (Fig. 4r). Quantification of *SOX3*, *BRA* and *GDF1* expression levels versus the relative position along the margin confirmed the significantly narrower expression of *GDF1* in attached versus detached epiblast halves (Fig. 4t). Yet *GDF1* expression, in contrast to tissue contraction (Fig. 4s), did not appear to have fully rescaled (compare the profiles in attached halves and controls in Fig. 4t). This prompted us to examine more closely the relationship between tissue contraction and gene expression. Reasoning that gene expression, responding more slowly than contractility, was likely to depend on the full deformation history of the tissue rather than its instantaneous contraction pattern, we plotted the *GDF1* level in small (approximately 100 µm) portions of the margin versus their integrated contraction (measured as the logarithm of their fold change in length; Fig. 4u). Whereas *GDF1* was approximately uniform in regions that had steadily stretched or contracted (corresponding to extremes of the integrated contraction), this analysis revealed an approximately linear relationship between integrated contraction and *GDF1* levels in regions with intermediate deformation histories (for example, cells in intact embryos that transition from the stretched to the contracting domain in the course of development). Notably, the data for the different conditions collapsed on the same curve (Fig. 4u (solid lines)), suggesting that integrated contraction is predictive of *GDF1* expression and its redirection after perturbations. Consistent with this, when we defined a ‘contracted domain’ based on thresholding the integrated contraction (Methods), its relative size closely predicted the relative size of the *GDF1* domain (Fig. 4v).

Note that, in these experiments, stages of development were precisely quantified according to the progression of gastrulation movements and matched between conditions to allow for an accurate comparison (confounding effects that might arise from the advection of gene expression territories can be ruled out; see Methods for details). Furthermore, when embryos with free edges were allowed to develop for longer, the entire marginal tissue contributed to a fully elongated primitive streak, leaving a largely depleted embryonic territory to contribute to ectoderm, as opposed to posterior halves with attached borders, which maintain a balance between the pools of cells that contribute to different territories (Extended Data Fig. 6 and Supplementary Video 6). Taken together, these experiments, in which only the mechanical boundary condition was changed, demonstrate that self-organized tissue contractility acts upstream of gene expression to enable the proper balancing and rapid rescaling of embryonic territories.

**Fig. 5:**
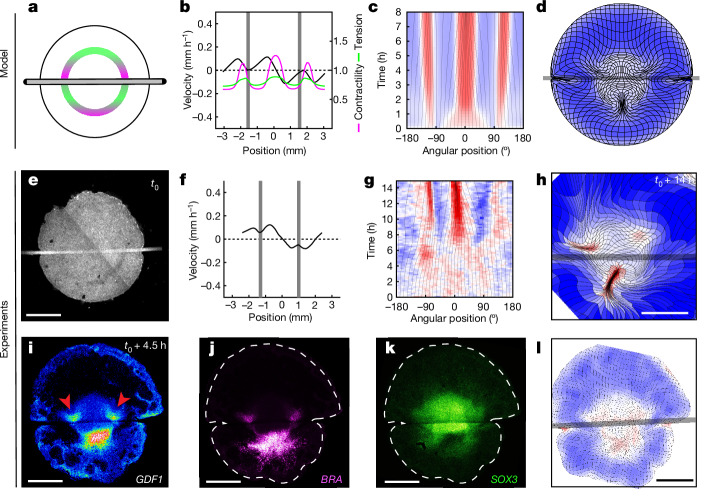
Mechanical friction redirects tissue motion/axis formation, inducing ectopic embryo formation. **a**–**h**, Model predictions (**a**–**d**) and the experimental response (**e**–**l**) to localized friction. **a**,**e**, Sketch of the experiment (**a**) and memGFP embryo with a hair on its ventral side (**e**). **b**,**f**, Profiles of contractility (magenta), tension (green) and velocity (black) along the margin at *t*_0_ + 4 h; 0 mm is posterior. **c**,**g**, Kymograph of margin strain rates; 0° is posterior. **d**,**h**, Deformation maps at 8 h (**d**) and 14 h (**h**) after hair deposition. *n* = 33 out of 34 biologically independent embryos. **i**–**l**, *GDF1* (**i**; the arrowheads point to ectopic expression), *BRA* (**j**) and *SOX3* (**k**) expression and the corresponding deformation map (**l**), 4.5 h after hair deposition (grey box). *n* = 15 biologically independent embryos. Colours in **c**, **d**, **g**, **h** and **l** quantify contraction (red) and expansion (blue) as in Fig. 1. Scale bars, 1 mm (**e** and **h**–**l**).

**Extended Data Fig. 7:**
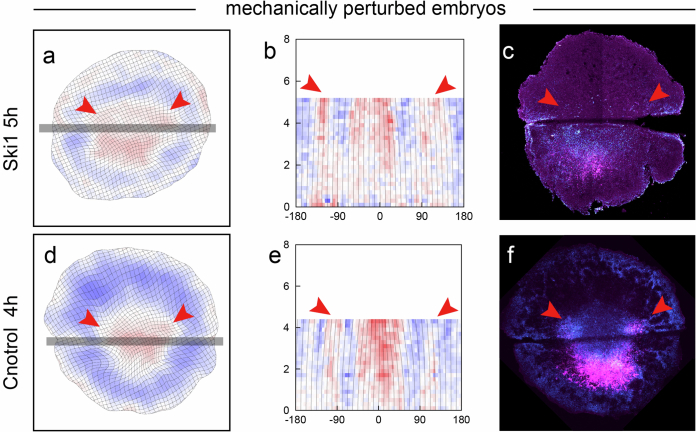
Inhibition of obstacle-induced ectopic embryo formation by Ski-1 treatment. **(a-f)** Ski-1-treated (a-c, n = 7 biologically independent embryos) and control (d-f, n = 10 biologically independent embryos) embryos on which a hair has been deposited. (a, d) Deformation maps. (b, e) Kymograph of strain rates at the margin. (c, f) Gdf1 (cyan) and Bra (magenta) expression. Red arrows point to sites of ectopic contraction anterior to the hair.

**Extended Data Fig. 8:**
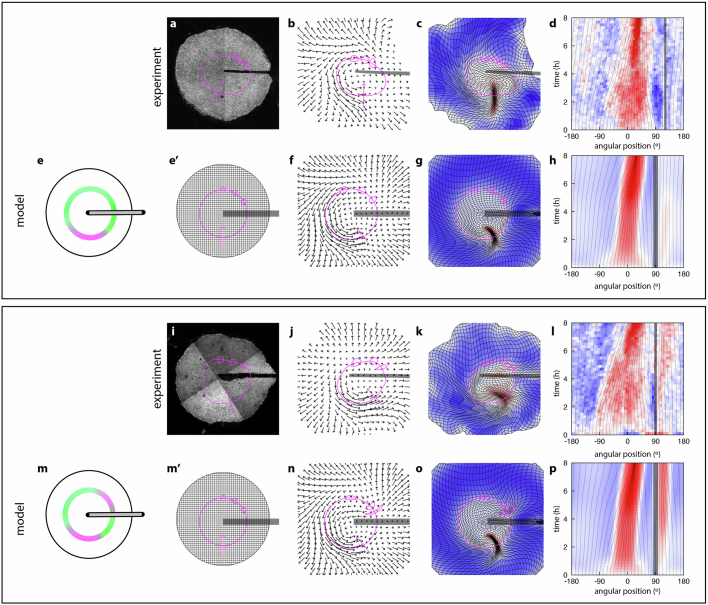
Asymmetric perturbations probe the range of tension propagation. (**a**-**h**) Long-range inhibition of ectopic contraction in the presence of an obstacle interfering with tension propagation one side of the margin in experiments (a-d; n = 5/7 biologically independent embryos) and simulations (e-h). Notice the propagation past the anterior and all the way to the obstacle of motion towards the posterior (velocity fields at t0 + 8 h in b,f) and stretching (deformation maps at t0 + 8 h in c, g, overlaid with initially circular contours from a,e’, and kymographs of strain rate along the margin in d,h). In simulations, this outcome is obtained with parameter values that increase the range of tension propagation by 20% compared to our main parameter set (cf. Supplementary Methods Bc). (**i-p**) Occasionally in experiments (i-l; n = 2/7 biologically independent embryos), and with our main parameter set in simulations (m-p), motion towards the posterior does not propagate all the way to the obstacle and an ectopic contraction develops anterior to the obstacle, as seen in the velocity fields (j,n), deformation maps (k,o), and kymographs of strain rate along the margin (l,p). Magenta arrows in the second (resp. third) column show the orientation of the presumptive axis at t0 (resp. t0 + 8 h), highlighting a rotation of the axis induced by the obstacle.

Mechanical self-organization provides a parsimonious account of the classic studies that initially revealed the regulative and self-organized nature of early avian development^2,3^. However, the physical cuts and separation that they involve remain invasive perturbations, and one cannot rule out a contribution of wound healing or other responses elicited at cut edges to experimental outcomes. On the other hand, if these outcomes indeed manifest mechanical self-organization, it should be possible to achieve similar effects through less-invasive mechanical perturbations interfering with tension propagation along the margin, such as localized friction. To introduce friction at the margin, a hair or a nylon wire was deposited across the epiblast (Fig. 5a,e). The hair or wire acted as an obstacle against the flow and slowed down tissue motion without provoking its complete arrest, indicating that it did not act as a tight barrier. As predicted by the model (Fig. 5b–d), two contraction foci rapidly emerged and elongated into primitive streaks in the anterior epiblast, just anterior to the hair (where tension propagation along the margin is obstructed), whereas the endogenous contracting domain in the posterior narrowed to accommodate the new mechanical boundaries of the margin (Fig. 5f–h). The ectopic primitive streaks were preceded by the emergence of *GDF1* expression (Fig. 5i,k) and accompanied by redirection of *SOX3*^+^ and *BRA*^+^ embryonic territories by 4.5 h after obstacle deposition (Fig. 5j,k). Ectopic embryo formation was impeded when embryos were cultured in the presence of Ski-1, although the margin contracted behind the obstacle as in the controls (Extended Data Fig. 7), confirming that the β-catenin mechanosensitive pathway underlies *GDF1* regulation in intact and in mechanically perturbed embryos. To further investigate the range of tension propagation, we deposited an obstacle across just one side of the epiblast (Extended Data Fig. 8). Most often in this condition, no ectopic contraction was observed: motion towards the posterior, and the accompanying stretching, propagated past the anterior and all the way to the obstacle, demonstrating the ability of tension to propagate at long range and inhibit embryo formation. However, occasionally, an ectopic contraction did form anterior to the obstacle, suggesting that the range of tension propagation is larger than needed to reach the anterior margin, but not very much so. This is fully consistent with our model: our choice of parameter values that are sufficient for inhibition in the anterior, but not much more, makes them marginal for the asymmetric condition. With our default parameters, an ectopic contraction forms, but a 20% change in a parameter affecting the range of tension propagation is sufficient to suppress it (Supplementary Methods B (paragraph c) and Extended Data Fig. 8).

Together, these perturbations demonstrate that mechanical tension propagates at long range along the margin to inhibit ectopic embryo formation, and that noninvasively interrupting its path through friction is sufficient to rapidly and persistently redirect force generation and tissue motion, leading to the reallocation of embryonic territories through the modulation of critical gene expression.

## Discussion

Hans Driesch originally defined regulation as a group of events that follow a disturbance and lead to a restoration towards the normal state of a living organism^1^, recognizing that this implied communication between its parts. Whereas Driesch could only speculate about the nature of this communication, and subsequent investigations mostly focused on molecular interactions, embryonic regulation was first evidenced through physical perturbations (the removal of parts of an embryo), and also implies a redirection of the mechanical forces driving morphogenesis. Our experiments identify this redirection of force generation and tissue motion as the earliest response to perturbations, prefiguring subsequent changes in gene expression. Indeed, the full pattern of tissue flows that shape the intact embryo and its response to perturbations are captured by a model that rests solely on mechanical feedback: the balance between local contractility and transmitted tension, which governs the local state of contraction or stretching of the tissue, feeds back on contractility itself, with contraction and stretching promoting and inhibiting contractility, respectively. As shown in Fig. 1f, contractility therefore locally self-activates and transmitted tension acts as an inhibitor. Regardless of any model, the demonstration that mechanical boundary conditions have a critical role in the redirection of tissue motion and gene expression (Fig. 4) unambiguously identifies mechanical forces as a major signal operating upstream of gene expression in embryonic self-organization. As the actively contracting domain of the margin adjusts its size in response to these forces, embryonic regulation can be understood as a form of mechanical homeostasis. Homeostasis cannot be restored, and the embryo fails to rescale, if it cannot regain traction on the vitelline membrane after a cut.

A role for mechanics in embryonic self-organization has long been proposed^16–18,31^. In one class of models, a pattern of forces emerges through the transport of contractile material, whether in the form of cell migration^16,17,31^ or advection of myosin by the cell cortex^32^. The rapid regulation that we observed in the early embryo is inconsistent with such models, as the timescale for advection is the timescale of the entire process by which the primitive streak is formed. In our model, mechanical tension instead acts as a rapidly propagating, long-range signal regulating contractility, as in a model proposed previously for cell polarization in embryonic epithelia^18^, although the output here is not a stable arrangement of cell states but a steady pattern of contractility and continuous tissue flow. Such a role for mechanics in self-organized developmental processes is increasingly evident^25,27,30^, yet how mechanical and molecular signals combine to organize patterning and morphogenesis at the tissue scale has remained unclear. Our findings indicate that it is the combination of both self-organizing and gene-regulatory features of mechanical forces that enables embryonic regulation. In this instance, they further suggest that the effect of mechanics on gene expression is relayed through the mechanosensitive β-catenin pathway. Supporting a coherent response to perturbations, contractility and *GDF1* expression respond in a similar manner to the same mechanical stimulus (contraction versus stretching), yet our observations suggest that the responses occur on distinct spatial scales (if force generation is regulated through the turnover of supracellular cables, when gene regulation requires the intracellular relaying of mechanical signals) and different timescales. Whereas the rapid turnover of the force-generating machinery can support a rapid modulation of force generation and tissue motion, our data indicate that, on the slower timescale of gene regulation, *GDF1* levels depend on the integrated deformation of the tissue.

Finally, we note that, although self-organized tissue mechanics can support spontaneous symmetry breaking, as manifested in ectopic embryo formation, it does not account for the original symmetry-breaking event in normal development. Indeed, *GDF1*, the expression of which is restricted to the posterior epiblast before gastrulation movements initiate, is itself required for primitive-streak morphogenesis and can redirect tissue motion when applied ectopically. Thus, tissue mechanics (at least, as we describe it here) functions as a canalizing feedback, operating in a time window of a few hours between the onset of gastrulation movements and the emergence of the fully formed streak, rather than as the initial trigger in normal axis formation. This illustrates the high level of interdependency between mechanical and molecular signals that safeguards development from deleterious deviations. Given the universal role of mechanical forces in shaping tissues, we anticipate that our findings will be relevant to patterning processes in diverse organisms, especially amniotes, including humans, which have a regulative and self-organized development.

## Methods

### Animals

All experimental methods and animal husbandry for transgenic quails were performed in accordance with the guidelines of the European Union 2010/63/UE, approved by the Institut Pasteur ethics committee authorization #dha210003, and under the GMO agreement 2432.

### Embryo imaging, orientation and laser dissection

Transgenic memGFP quail embryos^6^ were collected at stage XI using a paper filter ring and cultured on a semi-solid nutritive medium of thin chicken albumen, agarose (0.2%), glucose and NaCl, as described previously^6^. The embryos were then transferred to a glass-bottom six-well plate (Mattek) with 2 ml of the nutritive medium and imaged at 38 °C using the Zeiss LSM 900 microscope with ×2.5 or ×5 objectives. The time interval between two consecutive frames was 6 min. Sample size was not predetermined. The selection of embryos used in experiments was not randomized and blinding was not used for the analyses.

For bisection experiments, epiblasts were oriented by analysing tissue flows in real-time using PIV. After 1–2 h of image acquisition, the margin, the embryonic and extraembryonic territories, and the presumptive anterior–posterior axis of the embryo were determined automatically, as previously described^6^. Next, the spatial *x*–*y* coordinates of a line passing through the centre of the epiblast, with a specific angle to the anterior–posterior axis (as explained in Extended Data Fig. 4), were obtained and transferred to the ROE SysCon software of the laser dissector. Laser severing was performed using the UGA-42 firefly module coupled to a 355 nm pulsed laser (100% power) from Rapp Optoelectronic and the above-mentioned microscope and objectives. After bisection, the two halves of the embryo were moved on two separate vitelline membranes, or one half was left in its position and the other half was gently removed with a mouth pipette.

To ensure accurate comparison between posterior halves with fixed/free borders and intact epiblasts, their developmental stage was precisely matched. As described previously^6^, embryos were staged according to the integrated contraction of a posterior segment of the margin. This was evaluated on the fly based on PIV analysis of tissue motion. Embryos exhibiting an identical contraction (20%, reached in around 2 h) were bisected and allowed to develop until they contracted by the same amount (50%, reached in about 3 h). The equivalent stage for control (intact) embryos was determined by calculating the total contraction (before + after the cut) of posterior halves (60%; around 5 h). Thus, embryos presenting the same amount of tissue converging towards the primitive streak are being compared, ruling out possible confounding effects due to advection. For simplicity, hours only were reported in figures.

Attachment of the embryo was favoured by cooling down the embryo at room temperature for 1 h and removing excess liquid culture medium between the freshly cut edge and the vitelline membrane, whereas attachment was disfavoured by imaging posterior halves immediately after the bisection. Embryos were subjected to a second laser dissection if the edges reattached, as in Extended Data Fig. 5 and Supplementary Video 6.

### Pharmacological treatment and obstacle

For the drug delivery experiments, 0.8% DMSO (0.8% v/v), calyculin A (Tocris, 35 nM), H1152 dihydrochloride (Tocris, 25-75 µM) and Ski-1 (Tocris, 75-125 µM) were added to the culture medium. For the obstacle experiment, memGFP quail embryos were oriented by eye, and a fragment of hair, or a Nylon wire coated with Cell-Tak (diameter, ~100 µm; length, ~6,000 µm) was gently deposited onto the ventral side of the embryo.

### In situ hybridization and immunostaining

Quail embryos were fixed in ice-cold 4% formaldehyde, dehydrated in PBST (PBS/0.1% Tween-20) with increasing methanol concentrations (25%, 50%, 75% and 100%), and then rehydrated. Hybridization with DIG-labelled RNA probes was performed overnight at 65 °C in hybridization buffer (5× SSC pH 4.5, 50% formamide, 1% SDS, 50 mg ml^−1^ yeast tRNA, 50 mg ml^−1^ heparin). The next day, the embryos were thoroughly washed and treated for 6 h with a blocking solution of MABT, 2% BBR (Boehringer blocking reagent) and 20% lamb serum. The embryos were then incubated overnight with an AP-coupled anti-DIG antibody (Biotechne, MAB7520, 1:2,000) in the blocking solution and finally stained with NBT/BCIP liquid substrate (Sigma-Aldrich). After the staining, the embryos were temporarily mounted onto a slide and photographed at different magnification using the SteREO Discovery.V8 system (Zeiss; equipped with an AxioCam MRc (Zeiss)). For the DIG-labelled probe, an 807 bp fragment of the *SNAI2* coding sequence (from nucleotides 165 to 971) was PCR-amplified from quail cDNA and cloned into the pGEM-T Easy vector (Promega). The embryos were stained with antibodies against BRA (Biotechne, AF2085, 1:200).

### RNAscope

The samples were fixed overnight at 4 °C in ice-cold 4% formaldehyde and then washed in PBST for 6 h. Staining was performed using the RNAscope Multiplex Fluorescent Reagent Kit V2 according to the manufacturer’s instructions with the following modification for whole-mount staining of quail blastoderm embryos. For sample pretreatment, no retrieval or proteinase treatment was performed. The probes used for the stainings were as follows: RNAscope Probe Cja-*GDF1* (Bio-Techne, 593421), RNAscope Probe Cja-T-C2 (Bio-Techne, 587391-C2) and RNAscope Probe Cja-LOC107312850-C3 (Bio-Techne, 587381-C3), and were detected with the Opal520, Opal570 and Opal650 reagents (Perkin Elmer, 1:750 in TSA Buffer). The embryos were then mounted between the slide and coverslip using Fluoromount-G mounting medium (00-4958-02).

### Quantification of gene expression and contraction along the margin

To relate tissue deformation and gene expression, images of fixed samples were aligned with the last timepoint from live imaging. The RNA levels along the embryo margin (Fig. 4t,u and Extended Data Fig. 5g) were measured by integrating the maximum-*z*-projected signal across a 400-µm-wide strip centred on the margin. The levels were normalized for each embryo to ~1 and ~0 inside and outside, respectively, the expression domain. The instantaneous size of the contracting domain of the margin (Fig. 4s) was determined from the profile of tangential velocity along the margin (see Fig. 4f,n), as the interval between the extrema of the velocity. The integrated contraction of a margin portion (as in Fig. 4u and Extended Data Fig. 5g) was defined as the logarithm of its fold change in length over the course of the experiment, normalized to the logarithm of the target contraction for that experiment (as specified above, 50% reduction in length for epiblast halves and 60% reduction for intact epiblasts; the sign is chosen such that the normalized contraction is around −1 in the posterior). The relative sizes of the *GDF1* domain and contracted domain (Fig. 4v) were defined by thresholding the spatial profiles of *GDF1* and contraction (see Fig. 4t and Extended Data Fig. 5g); the thresholds were taken to correspond to the midpoint of the approximately linear relationship between contraction and *GDF1* in Fig. 4u (normalized expression >1/2, normalized contraction <−1/4).

### Model

We considered a hierarchy of models to explore self-organization of force generation along the embryo margin through feedback of tissue tension on contractility. In the simplest instance, the margin is described as a 1D tensile line with a fixed length and periodic boundary conditions, and the surrounding tissue is ignored, corresponding to a limit at which force transmission along the margin dominates over force transmission to the surrounding tissue. We can further take a limit at which advection along the margin is negligible, which is the relevant regime for the embryo (regulation occurs on timescales that are shorter than the characteristic timescale for advection, which is the timescale over which the primitive streak emerges).

In the context of this minimal model, we identify contractility with the active tension *T*_a_ generated at the margin. Assuming that this active tension combines with a linear viscous resistance to stretching, the total tension *T*(*s*) at a point *s* along the margin is given by

$$T(s,t)={T}_{{\rm{a}}}({\rm{s}},{\rm{t}})+\nu \dot{{\varepsilon }}({\rm{s}},{\rm{t}})$$

where $\dot{\varepsilon }$ denotes the strain rate (the local elongation rate of the margin) and *ν* is a 1D viscosity. Assuming that the active tension varies in response to the strain rate, we write

$$\frac{{\rm{\partial }}{T}_{{\rm{a}}}}{{\rm{\partial }}t}+u\frac{{\rm{\partial }}{T}_{{\rm{a}}}}{{\rm{\partial }}s}=\frac{1}{\tau }\left[{T}_{0}\left(1+\zeta \,{\rm{t}}{\rm{a}}{\rm{n}}{\rm{h}}\left(\alpha -\beta \frac{v}{{T}_{0}}\dot{{\varepsilon }}\right)\right)-{T}_{{\rm{a}}}\right]+D\frac{{{\rm{\partial }}}^{2}{T}_{{\rm{a}}}}{{\rm{\partial }}{s}^{2}}$$

where *τ* is a characteristic timescale for regulation, and we have included a diffusion term with diffusivity *D* to account for non-local self-activation of contractility, which might arise from the recruitment of neighbouring cell–cell junctions into cables that span several cells and are constantly turning over within the margin.

With the surrounding tissue being neglected, mechanical balance implies that the tension *T* is uniform along the margin, and conservation of the total margin length implies that the average strain rate vanishes, $\left\langle \dot{\varepsilon }\right\rangle =0$, so that

$$\frac{{\rm{\partial }}{T}_{{\rm{a}}}}{{\rm{\partial }}t}+u\frac{{\rm{\partial }}{T}_{{\rm{a}}}}{{\rm{\partial }}s}=\frac{1}{\tau }\left[{T}_{0}\left(1+\zeta \tanh \left(\alpha +\beta \frac{{T}_{{\rm{a}}}-\langle {{\rm{T}}}_{{\rm{a}}}\rangle }{{T}_{0}}\right)\right)-{T}_{{\rm{a}}}\right]+D\frac{{{\rm{\partial }}}^{2}{T}_{{\rm{a}}}}{{\rm{\partial }}{s}^{2}}$$

Thus, active tension is upregulated and downregulated where it is above and below, respectively, its spatial average. Mathematically, this model is equivalent to a molecular activator–inhibitor model, in which the tension *T* = ⟨*T*_a_⟩ serves as a long-range inhibitor, in the limit of infinitely fast and long-range inhibitor diffusion. If the coefficient *β* representing the strength of mechanical feedback is large enough, the model supports spontaneous symmetry breaking and the stable maintenance of regions of high and low contractility. In the relevant parameter regime, the steady state of the model is governed by the motion of narrow fronts between these regions that tend towards invariant proportions, corresponding to a fixed total tension. When advection is taken into account, the fronts are displaced to a position where advection is balanced by the tendency to return to this preferred, homeostatic tension. Details of the model and its analysis are provided in the Supplementary Discussion.

Our full 2D model incorporates the same mechanical regulation of contractility into our previously described fluid-mechanical model of tissue flows in the embryo^6^. In brief, the embryonic disk is described as a 2D viscous fluid driven by tension along the margin, with a prescribed divergence term *γ* that allows for non-uniform areal expansion of the embryonic disk. The profile of stresses imparted on the tissue by the margin is assumed to keep an invariant, Gaussian profile, such that distributed force generation at the margin and its regulation can still be described in terms of a 1D profile of contractility. To allow for a nonlinear relationship between mechanical load on cell–cell junctions and contraction rate, and a saturation of the contraction rate, the margin is explicitly described as a 1D viscoelastic line; the rest length *l*_0_ of an element of margin varies as

$$\frac{1}{{l}_{0}}\frac{{\rm{d}}{l}_{0}}{{\rm{d}}t}=\lambda {\dot{{\varepsilon }}}_{0}W\left(\frac{T}{c{T}_{{\rm{s}}}};\lambda \right)+\frac{\gamma (x,t)}{2}$$

where *c* denotes the contractility (which is no longer identified with an active tension but could be understood to represent the local density of active myosin or supracellular cables), *W* is a nonlinear saturating ‘walking kernel’ (see ref. ^24^) and the term *γ*/2 is included to allow for the shrinking of cables through cell ingression (corresponding to *γ* < 0) at the primitive streak (see ref. ^6^). *T*_s_ denotes a stall force per unit contractility at which junctions transition from contraction to yielding, and the parameter *λ* controls the nonlinearity of the walking kernel. With *E* denoting the elastic modulus of the margin, we obtain the system of equations

$$\begin{array}{c}\frac{{\rm{\partial }}c}{{\rm{\partial }}t}+u\frac{{\rm{\partial }}c}{{\rm{\partial }}s}=\frac{1}{\tau }\left[{c}_{0}+\Delta c\,\tanh \left(\alpha -\frac{\beta \dot{{\varepsilon }}}{\lambda {\dot{{\varepsilon }}}_{0}}\right)-c\right]+D\frac{{{\rm{\partial }}}^{2}c}{{\rm{\partial }}{s}^{2}}\\ \frac{{\rm{\partial }}T}{{\rm{\partial }}t}+u\frac{{\rm{\partial }}T}{{\rm{\partial }}s}=E\left[\dot{{\varepsilon }}-\frac{\gamma }{2}-\lambda {\dot{{\varepsilon }}}_{0}W\left(\frac{T}{c{T}_{{\rm{s}}}};\lambda \right)\right]\end{array}$$

where *T* is the elastic tension along the margin, which determines the velocity *u* through the Stokes equation describing tissue motion.

The prescribed area changes, which approximate experimentally observed area changes using analytic functions according to a previous study^6^, include contributions from expansion of extraembryonic tissue and ingression at the primitive streak. Here, the contribution of ingression at the primitive streak is included when modelling the intact epiblast (Fig. 1), to most closely compare with the model from ref. ^6^ in which both area changes and active forces were prescribed, as well as for the asymmetric perturbations in Extended Data Fig. 8, which have a limited effect on primitive streak formation. On the other hand, this contribution is omitted in simulations of posterior halves (Fig. 4) and with a full obstacle (Fig. 5), as we do not wish to explicitly model the redirection of cell ingression when primitive streak formation is displaced; this is inessential for our purposes, as our focus in on the redirection of force generation and tissue flows upstream of primitive streak formation, and as ingression makes a limited contribution to shaping the embryo in the time interval considered here, as quantified previously^6^.

This 2D model is used to simulate the full course of margin regulation and tissue flows within the embryonic disk upon perturbations, and in a simplified geometry is amenable to a similar analytical description as the minimal 1D model. For numerical simulations, it was implemented in Python, using the FEniCS finite element platform^33,34^. A detailed discussion is provided in the Supplementary Methods.

### Reporting summary

Further information on research design is available in the Nature Portfolio Reporting Summary linked to this article.

## Online content

Any methods, additional references, Nature Portfolio reporting summaries, source data, extended data, supplementary information, acknowledgements, peer review information; details of author contributions and competing interests; and statements of data and code availability are available at 10.1038/s41586-024-07934-8.

## Supplementary information

Supplementary Information Supplementary Discussion, Supplementary Methods, Supplementary Tables 1 and 2 and Supplementary References

Reporting Summary

Supplementary Video 1 A model for self-organized force generation at the embryo margin. Top, transmitted light (left) and GFP fluorescence (right) video of a quail embryo depicting the embryonic and extraembryonic territories and the motion of the tissue. Left, trajectories (the overlay denotes the embryo margin); velocity profiles at the margin and 2D deformation from an average of 6 different embryos between *t*_0_ and *t*_0_+8h; Right, mechanical self-organized model for the regulation of tissue contractility at the margin, with predicted contractility (magenta), tension (green) and velocity profiles (black) at the margin and 2D deformation

Supplementary Video 2 Tissue contractility modulates *GDF1* expression in intact epiblasts. The effect of calyculin A, H1152 and Ski-1 on tissue motion and *GDF1* and *BRA* expression.

Supplementary Video 3 Self-organized tissue mechanics drives ectopic embryo formation after epiblast subdivision. Top, model predictions for the response to epiblast bisection in anterior halves showing profiles of contractility (magenta), tension (green) and velocity (black) at the margin and 2D deformation. Bottom, experimental bisections showing initial image acquisition, 2D deformation and corresponding expression of *GDF1* and *SNAI2* in halves with or without epiboly and treated with H1152.

Supplementary Video 4 The procedure for monitoring embryo formation after epiblast bisection. Intact embryos were imaged for 1–2 h and analysed using PIV on the fly. The orientation of the cut was defined following the automated detection of the margin and anteroposterior axis. The coordinates of the desired cut were transferred to the laser-dissection system. Bisected and separated epiblast halves were then fixed at early (~5 h) or late (>10 h) timepoints and the expression of *GDF1* and *SNAI2* was then verified.

Supplementary Video 5 Mechanical feedback (re)scales embryonic territories. Top, model predictions and experimental response to fixed and free boundary conditions in posterior epiblast halves after 3 h. Contractility, tension and velocity profiles are shown in magenta, green and black, respectively. Bottom, expression of *GDF1*, *BRA* and *SOX3*.

Supplementary Video 6 The long-term effect of mechanical boundary on embryonic territories in posterior halves. Left, posterior half with attached borders, imaged for 8 h. Right, posterior half with repeated laser cuts to release border attachment, imaged for 8 h. Note the unbalanced contribution of the entire margin to the forming primitive streak at the expense of the epiblast territory.

Supplementary Video 7 Mechanical friction redirects tissue motion, inducing ectopic embryo formation. Top, model predictions for localized friction across the epiblast showing contractility (magenta), tension (green) and velocity profiles (black) at the margin and 2D deformation. Bottom, memGFP embryo with a hair on its ventral side showing velocity profiles at the margin and 2D deformation maps at *t*_0_+14h and *t*_0_+4.5h with corresponding *GDF1*, *BRA* and *SOX3* expression.

## Source data

Source Data Fig. 4

Source Data Extended Data Fig. 2

## Data Availability

The datasets presented in this study are available at Figshare (10.6084/m9.figshare.26004184)^35^. Source data are provided with this paper.

## Code availability

Custom code used to simulate the epiblast using finite elements is available at GitHub (https://github.com/achamolly/mechanical_self_organisation/). A detailed description of its functionality is provided in the Supplementary Methods.
