# Supplementary material for: Self-organized tissue mechanics underlie embryonic regulation
Source: Nature. 2024 Sep 11;633(8031):887–94. doi: 10.1038/s41586-024-07934-8 (PMC11424473; doi:10.1038/s41586-024-07934-8)
Supplement: Supplementary file 1 — Supplementary Discussion, Supplementary Methods, Supplementary Tables 1 and 2 and Supplementary References [file 41586_2024_7934_MOESM1_ESM.pdf]

nature portfolio

https://doi.org/10.1038/s41586-024-07934-8

Supplementary information

Self-organized tissue mechanics underlie embryonic regulation

In the format provided by the authors and unedited

Supplementary Notes for "Self-organized tissue mechanics underlie embryonic regulation"

Paolo Caldarelli, Alexander Chamolly,* Aurélien Villedieu,* Olinda Alegria-Prévot, Carole Phan, Jerome Gros,† and Francis Corson‡

CONTENTS

I. Supplementary Discussion 2
A. Overview of the theoretical models 2
B. 1D model of mechanical regulation along margin 4
1. Derivation 4
2. Predictions made by the 1D model 5
C. Non-linear 2D model with surrounding tissue 8
1. Derivation 8
2. The 1D model as a limiting case 12
3. New predictions 13

II. Supplementary Methods 14
A. Numerical simulations – Implementation 14
B. Numerical simulations – Choice of parameter values and boundary conditions 15

Supplementary References 16

III. Supplementary Tables 17

* Equal contribution
† jgros@pasteur.fr
‡ corson@phys.ens.fr

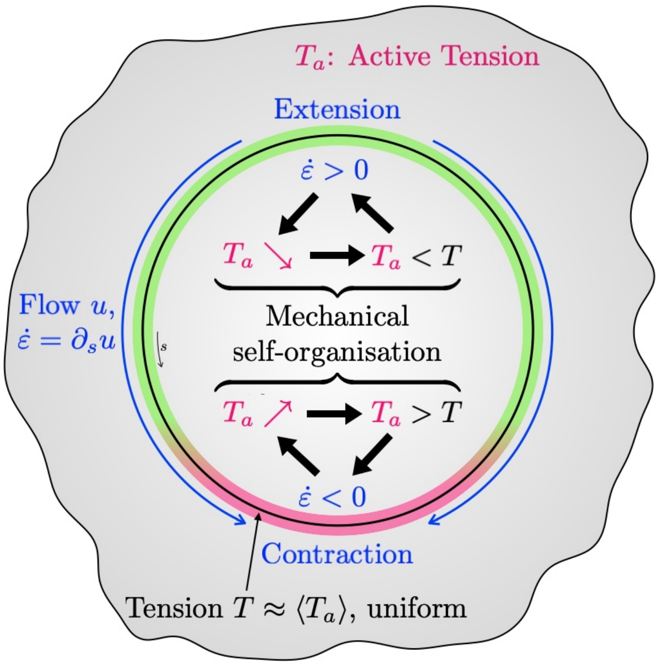

Supplementary Figure 1: Conceptual sketch of the 1D theoretical model. The margin is under tension, which is approximately uniform by force balance. In regions with elevated contractility, biologically generated active tension T_a wins over passive material stresses, leading to a contraction. This triggers a biological response, further increasing active tension in a positive feedback loop. Since the margin forms a closed ring, tension builds up along the entire length as the contraction develops in the posterior. This stabilises the posterior from perturbations that might lead to ectopic contractions.

I. SUPPLEMENTARY DISCUSSION

A. Overview of the theoretical models

a. 1D minimal model of margin regulation. In this article we refer to two versions of a theoretical model to describe the mechanical self-organisation of contractions along the circular margin between the embryo proper (EP) and the extra-embryonic tissue (EE). The first is a one-dimensional model that focuses on the regulation along the margin and ignores the surrounding tissue. It contains the key ingredients that enable the self-regulation, but excludes complications that are added in the second model to allow for quantitative comparison with experiments. It is, however, simpler to understand conceptually and analyse mathematically, and we thus retain it for illustration. Its variable is the biologically generated active tension T_a, which in dimensionless form is governed by the equation

$$\underbrace{\frac{\partial T_a}{\partial t}}_{\substack{\text{change in}\\ \text{active tension}}} + \underbrace{\mathcal{P}\, u \frac{\partial T_a}{\partial s}}_{\substack{\text{transport by}\\ \text{tissue flows}}} = \underbrace{1 + \zeta \tanh\left[\alpha + \beta\left(T_a - \langle T_a \rangle\right)\right] - T_a}_{\text{tension regulation}} + \underbrace{\mathcal{D} \frac{\partial^2 T_a}{\partial s^2}}_{\substack{\text{spatial extent}\\ \text{of cables}}} . \tag{1}$$

Here u denotes the tissue velocity. Changes in active tension comprise three contributions: advection, diffusion, and biological regulation. Advection is caused by transport of material parallel to the margin by tissue flows. The diffusion term models phenomenologically the spatial extent of cables, ensuring that spatial variations in contractility cannot be arbitrarily sharp. The regulation term builds in our hypothesis of mechanical feedback on contractility, whereby tension inhibits contractility. As a generic form to implement a smooth transition between up- and downregulation, we assume a sigmoidal dependence on the local strain rate, which itself depends on the difference between the local value of T_a and its average along the margin, $\langle T_a \rangle$. The parameters α, β, and ζ control the location, slope, and amplitude of the transition, respectively, while $\mathcal{P}$ and $\mathcal{D}$ denote the relative importance of advection and diffusion on the time scale of self-regulation, and are small. Although a detailed model of the supracellular cables that make up the margin is beyond the scope of this study, we note that the regulation and diffusion terms entering Eq. (1) would naturally arise if the turnover of cables depends on the local strain rate (i.e., stretching promotes their breakdown)

and if cables grow through the recruitment of neighboring junctions (such that contractility spreads). Further details and a derivation may be found in section I B 1, and an illustration is given in Supp. Fig. 1.

A key prediction of this model is an instability that gives rise to a bi-contractile state with a single contracting and a single extending region that occurs when the condition

$$\beta > \frac{1 + 4\pi^2 \mathcal{D}}{\zeta \operatorname{sech}^2 \alpha}, \tag{2}$$

corresponding to a sufficiently sensitive regulation, is satisfied. The proportion of the contractile domain, ρ, is approximated by the expression

$$\rho \approx \frac{1}{2} + \frac{\alpha}{2\beta\zeta}, \tag{3}$$

(more accurately given in Eq. (24b)), which is independent of the margin length. As a result, the model predicts a rescaling of the contractile domain in the posterior half after ablation. A requirement for this is the reattachment of the cut to the underlying vitelline membrane, since this allows tension in the margin to be sustained as in the intact embryo. Likewise, an ablated anterior half is predicted to develop the same instability if the cut reattaches. However, since an initial bias in contractility determines the location of the contraction, and contractility in the anterior half is maximised at the cut, in this case two smaller ectopic contractions may occur. When the cut does not reattach, no tension can be sustained in the margin and the model predicts the formation of a single contractile domain, as observed in experiments. Further details of the predictions of this model can be found in section I B 2.

b. 2D non-linear model with tissue flow. The second model is an extended version that additionally incorporates the dynamics of the surrounding tissue, as well as a more detailed, non-linear treatment of the tension regulation in the margin. Its variables are the contractility, c, and the margin tension, T, and the governing equations for the evolution along the margin are

$$\frac{\partial c}{\partial t} + \mathcal{P}\, u \frac{\partial c}{\partial s} = 1 + \zeta \tanh\left[\alpha - \beta\dot{\epsilon}\right] - c + \mathcal{D}\frac{\partial^2 c}{\partial s^2}, \tag{4a}$$

$$\frac{\partial T}{\partial t} + \mathcal{P}\, u \frac{\partial T}{\partial s} = \mathcal{E}\left[\dot{\epsilon} - W\left(\frac{T}{c};\lambda\right)\right]. \tag{4b}$$

where $W(x;\lambda) = \lambda^{-1}\tanh\lambda\,(x-1)$ is a non-linear "walking kernel". The quantities u and $\dot{\epsilon}$ refer to tissue velocity and extension rate tangential to the margin and are themselves determined through solving the Stokes equations for 2D viscous flow in the whole embryo,

$$-\boldsymbol{\nabla} p + \mathcal{T}\nabla^2 \boldsymbol{u} + \boldsymbol{\nabla}\cdot\boldsymbol{\sigma}_a = \boldsymbol{0}, \quad \boldsymbol{\nabla}\cdot\boldsymbol{u} = \gamma, \tag{5}$$

where $\boldsymbol{\sigma}_a$ is an active stress distribution that links gradients in tension to tissue flow, and $\gamma(\boldsymbol{x}, t)$ represents prescribed area changes to model tissue growth and ingression. In numerical simulations of the 2D model, the locus of the margin is furthermore not fixed, but it is advected with the instantaneous tissue velocity $\boldsymbol{u}$ acquired from solving the Stokes equations. This version of the model introduces three additional dimensionless parameters, the margin stiffness $\mathcal{E}$, the tissue viscosity $\mathcal{T}$, and λ, which parametrises the non-linearity of the walking kernel. Further details and a derivation may be found in section I C 1.

The 1D minimal model is recovered in the limit of a very stiff margin and very weakly viscous fluid, corresponding mathematically to the double limit $\mathcal{E} \gg 1 \gg \mathcal{T}$, when small perturbations of tension T and contractility c about a steady uniform state are considered (so that the walking kernel can be linearised). In this case, the tension is uniform to leading order and equal to the average contractility, which can then be identified with active tension up to a constant of proportionality. Further details about the connection between the models may be found in section I C 2.

The main purpose of the 2D model is to enable the numerical simulation of regulation in an embryo *in silico*, taking into account secondary effects such as epiboly and deformations, and compare with experiments. However, it also leads to some refined theoretical predictions. Most importantly, the competition between local regulation in the margin and non-local force transmission through the surrounding tissue determines the number of ectopic contractions. A linear stability analysis shows that the most unstable perturbation mode is

$$n \approx \max\left(1, \frac{\beta \operatorname{sech}^2 \alpha\mathcal{T}}{4\pi^3 \mathcal{D}}\right), \tag{6}$$

which implies that a high viscosity of the tissue favours ectopic contractions. Conversely, this suggests that a significant distinction in mechanical properties of the margin through the presence of supra-cellular actomyosin cables is conducive to the controlled formation of a single contraction. Likewise, the regulation sensitivity β needs to be sufficiently strong to generate an instability, yet a too large value also leads to ectopic contractions. More details and predictions can be found in section I C 3.

B. 1D model of mechanical regulation along margin

1. Derivation

We consider the dynamics of the margin in isolation and ignore the influence of the surrounding tissue. Since the margin width is narrow compared to its curvature and length, we ignore any cross-sectional variations and consider a one-dimensional line, parametrised by arc length s. Along the margin we allow for tangential tissue motion with velocity $u(s,t)$, which may also vary with time t, and define the contraction rate $\dot{\epsilon}(s,t) = \partial u/\partial s$. The contraction rate is positive when the margin is extending, and negative when it is contracting. We define $T(s,t)$ to be the tension of the margin. This is composed of a passive contribution due to internal dissipation, which we take to be effectively viscous with a one-dimensional viscosity ν, $T_p(s,t) = \nu\dot{\epsilon}$, and a biologically active tension (or contractility) $T_a(s,t)$, which responds to mechanical cues. We model this regulation with an advection-diffusion equation that includes a forcing term that depends on the extension rate $\dot{\epsilon}$,

$$\frac{\partial T_a}{\partial t} + u\frac{\partial T_a}{\partial s} = \frac{1}{\tau}\left[T_0\left(1+\zeta\tanh\left(\alpha - \frac{\nu\beta}{T_0}\dot{\epsilon}\right)\right) - T_a\right] + D\frac{\partial^2 T_a}{\partial s^2}, \tag{7}$$

where τ is the time scale of the regulation, T_0 a reference scale for the tension, ζ a dimensionless parameter indicating the amplitude of the regulation, α a dimensionless parameter controlling the crossover point in the response of active tension to stretching, β expresses the sensitivity of the regulation to stretching, and D is a diffusion coefficient that is added to phenomenologically incorporate a slightly non-local activation, as might be due to the recruitment of neighbouring cell-cell junctions into cables that span several cells and undergo constant turnover within the margin.

In the absence of advection or diffusion, Eq. (7) describes the relaxation of the active tension to a steady state $T_a = T_0\left(1+\zeta\tanh\left(\alpha - \beta\nu\dot{\epsilon}/T_0\right)\right)$. The model hence captures the idea that (for positive β) active tension is downregulated in extending regions of the margin ($\dot{\epsilon} > 0$) and upregulated in contracting regions ($\dot{\epsilon} < 0$), as could be due to a tension-dependent breakdown of the cables.

In order to close the model, we need to describe additionally how the contraction rate responds to stresses in the tissue. This is obtained by starting from the definition of tension,

$$T = T_a + \nu\dot{\epsilon}. \tag{8}$$

To proceed, we exploit the fact that for the minimal model we are considering the margin in isolation from the tissue, which amounts to neglecting friction with the surrounding tissue in relation to the internal stresses within the margin. Mechanical balance then implies that the total tension is uniform in space and varies only with time,

$$T(s,t) = T(t). \tag{9}$$

If the ends of the margin are closed in a loop (as in the case of the intact embryo), or fixed (as in the case of an ablated embryo where the cut has reattached to the vitelline membrane), the margin can sustain a non-zero tension $T > 0$. In contrast, when the ends are free (no reattachment) then $T = 0$. In the former case we can determine the value of T by considering the average of Eq. (8) over the length of the margin. Since the ends are fixed, so is the overall length and therefore the average contraction rate must be zero. Hence

$$T = \langle T_a \rangle, \tag{10}$$

where angled brackets indicate a spatial average, $\langle\cdot\rangle = L^{-1}\int_0^L \cdot\, \mathrm{d}s$, where L describes the length of the margin. Thus, the uniform total tension is equal to the average of the active tension, and the contraction rate is given by

$$\dot{\epsilon} = \frac{T - T_a}{\nu} = \frac{\langle T_a\rangle(t) - T_a(s,t)}{\nu}. \tag{11}$$

The tissue thus contracts (respectively extends) when the local value of the active stress is above (respectively below) its average value along the margin (see Supp. Fig. 2 for an illustrative example). Within the framework of this model,

mechanical tension thus functions as a signal that inhibits contractility at a distance from the regions where it is highest, akin to the diffusing inhibitor in a molecular activator-inhibitor model, although on the long time scales of development, the transmission of mechanical forces is near instantaneous.

Combining Eqs. (7) and (11), we find that a single non-local differential equation governs the evolution of the active tension. We can gain further insight by scaling variables to make all parameters dimensionless. To this end, time is scaled by τ, stresses by T_0, and lengths by L, while the contraction rates are scaled by T_0/ν and velocities by $T_0 L/\nu$. The governing equation then reads

$$\boxed{\frac{\partial T_a}{\partial t} + \mathcal{P}\, u \frac{\partial T_a}{\partial s} = 1 + \zeta \tanh\left[\alpha + \beta\left(T_a - \langle T_a \rangle\right)\right] - T_a + \mathcal{D}\frac{\partial^2 T_a}{\partial s^2}}, \tag{12}$$

where two new dimensionless parameters are defined as

$$\mathcal{P} = \frac{\tau}{\nu/T_0}, \quad \mathcal{D} = \frac{D\tau}{L^2}. \tag{13}$$

The dimensionless quantity $\mathcal{P}$, which determines the importance of the advection term $u\partial_s T_a$ in Eq. (12), is the ratio of the active tension regulation time scale τ to the time scale ν/T_0 of advection of cables moving with the tissue. Since the experiments show that mechanical regulation occurs on the scale of minutes or a few dozens of minutes, while the tissue flows occur on the scale of hours, this number $\mathcal{P}$ is small. Similarly, the effect of non-local activation is limited to scales much shorter than the overall length of the margin, and so $\mathcal{D} \ll 1$. This leaves the minimal model with the governing equation (12) above and **five dimensionless parameters**, α, β, ζ, $\mathcal{P}$ and $\mathcal{D}$.

2. Predictions made by the 1D model

a. Instability of the uniform state. We note that Eq. (12) allows for a trivial solution,

$$T_a \equiv T = 1 + \zeta \tanh(\alpha), \quad \dot{\epsilon} = 0, \tag{14}$$

corresponding to a state with no motion and uniform tension $T = 1 + \zeta \tanh(\alpha)$. Spontaneous segregation into extensile and contractile regions occurs when this uniform state is unstable. In order to examine stability, we consider an infinitesimal perturbation $T_a = T + \eta \exp\left(2\pi i n s + \sigma_n t\right)$ with $\eta \ll 1$ and $n \geq 1$ indicating the mode of the perturbation. Substitution into Eq. (12) yields

$$\sigma_n = \zeta\beta \operatorname{sech}^2 \alpha - 1 - 4\pi^2 \mathcal{D} n^2. \tag{15}$$

Hence the most unstable mode is the first, $n = 1$, corresponding to a single contraction, and the condition for instability is

$$\boxed{\zeta\beta \operatorname{sech}^2 \alpha > 1 + 4\pi^2 \mathcal{D}} \tag{16}$$

We conclude that for an instability to occur it is necessary that the regulation amplitude ζ and the regulation sensitivity β are sufficiently large. Strong biases in the regulation, corresponding to large absolute values of α, discourage an instability. Diffusion acts to suppress a short wavelength instability (below a length scale $\sqrt{D\tau}$), but a too great value suppresses the instability altogether. By definition of ζ as the amplitude of contractility variation between posterior and anterior, we have $0 < \zeta < 1$ and hence $0 < \zeta \operatorname{sech}^2 \alpha < 1$. In practice, this condition requires that the sensitivity β is quite large, and we shall make the approximation $\beta \gg 1$ in what follows.

b. Phenomenology of the bi-contractile steady state. In numerical simulations, the model as described by Eq. (12) is generically observed to converge to a bi-contractile state with one contracting and one expanding region when the instability condition Eq. (16) is satisfied. An illustration is given in Supp. Fig. 2.

In steady state $\partial_t = 0$ and from Eqs. (11) and (12) it follows that

$$T = 1 + \zeta \tanh\left(\alpha - \beta\dot{\epsilon}\right) + \dot{\epsilon} + \mathcal{P}u\frac{\partial \dot{\epsilon}}{\partial s} - \mathcal{D}\frac{\partial^2 \dot{\epsilon}}{\partial s^2}. \tag{17}$$

Here the tension T on the left-hand side is uniform along the margin, while the right-hand side is a function of the extension rate. Thus, if multiple values of the extension rate $\dot{\epsilon}$ satisfying Eq. (17) exist, then the system allows for

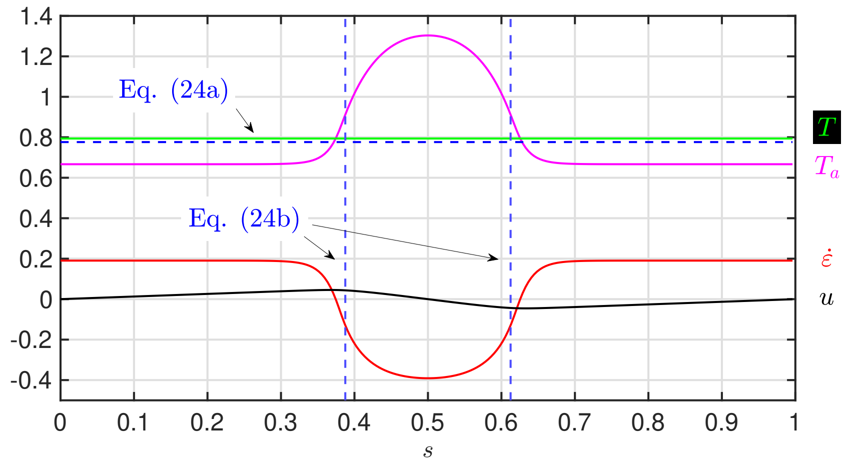

Supplementary Figure 2: Illustration of the steady state predicted by the minimal model. The vertical axis displays tension T (green), active tension T_a (magenta), extension rate $\dot{\epsilon}$ (red) and velocity u (black). All quantities are scaled to be dimensionless. Blue dashed lines indicate theoretical predictions for the size of the contractile domain ρ, Eq. (24b), and the tension T, Eq. (24a), using the value $u(s_f) = 0.0454$ obtained from the simulation to determine the Péclet number Pe. Simulation parameters as in Table 1: $\alpha = -1$, $\beta = 16$, $\zeta = 0.33$, $\mathcal{D} = 7.5 \times 10^{-4}$, $\mathcal{P} = 0.67$.

the formation of separate steady regions with different $\dot{\epsilon}$. Away from fronts, the extension rate is observed to be approximately uniform, and so the advection and diffusion terms become negligible. It follows that

$$T \approx 1 + \zeta \tanh\left(\alpha - \beta \dot{\epsilon}_{\pm}\right) + \dot{\epsilon}_{\pm}, \tag{18}$$

where $\dot{\epsilon}_{\pm}$ denotes possible values for the extension rate. Then, a bi-contractile state with both contracting ($\dot{\epsilon} = \dot{\epsilon}_{-} < 0$) and expanding ($\dot{\epsilon} = \dot{\epsilon}_{+} > 0$) regions is guaranteed to exist if the instability criterion Eq. (16) is satisfied, and the system converges to it on a time scale $\sim \tau$.

c. Fronts in the steady state. In order to gain further physical insight into this steady non-linear state, it is useful to consider first the nature of the contractility profile at the fronts between extensile and contractile regions.

Without loss of generality we consider a front at $s = 0$ with extension for $s < 0$ and contraction for $s > 0$. The centrepoint of the front is defined to be the inflection point of the regulation term, i.e. such that $\dot{\epsilon}(s = 0) = \alpha/\beta$. Assuming a high sensitivity, $\beta \gg 1$, we may treat the regulation as approximately bimodal, with $T_a \to 1 - \zeta$ in the extensile and $T_a \to 1 + \zeta$ in the contractile region. This implies that the same is true for the extension rates,

$$\dot{\epsilon}_{\pm} \approx T - 1 \pm \zeta, \tag{19}$$

with a so far undetermined value of the tension. Eq. (12) then reduces to the steady one-dimensional advection-diffusion equation with piecewise constant forcing,

$$0 = \begin{cases} 1 - \zeta - T_a - \mathcal{P} u \partial_s T_a + \mathcal{D} \partial_{ss} T_a, & s < 0, \\ 1 + \zeta - T_a - \mathcal{P} u \partial_s T_a + \mathcal{D} \partial_{ss} T_a, & s > 0. \end{cases} \tag{20}$$

Assuming that the fronts are narrow compared to the length of the domain (consistent with velocity profiles in experiments), the velocity u varies only slightly across the width of the front and we can approximate its value by a constant $u \approx u_0$. The solution for the active tension is then

$$T_a = \begin{cases} 1 - \zeta + \frac{2\zeta l_-}{l_- + l_+} \exp\left(s/l_-\right), & s < 0, \\ 1 + \zeta - \frac{2\zeta l_+}{l_- + l_+} \exp\left(-s/l_+\right), & s > 0, \end{cases} \tag{21}$$

where

$$l_{\pm} = \sqrt{\mathcal{D}} \left(\sqrt{1 + \mathrm{Pe}^2} \pm \mathrm{Pe} \right), \quad \mathrm{Pe} = \frac{u_0 \mathcal{P}}{2\sqrt{\mathcal{D}}}. \tag{22}$$

Here we define a Péclet number Pe that measures the relative importance of advection and diffusion in shaping the front. The value of u_0 and hence Pe is to be determined *a posteriori* from the requirement that the fronts are stationary.

We see that passive regulation along the margin forms the profile of the front into different shapes upstream and downstream. The upstream length l_- is compressed, while the downstream length l_+ is extended when $\mathcal{P} > 0$. The overall dimensionless width of the front $l_+ + l_-$ scales as $\sqrt{\mathcal{D}}\sqrt{1+\mathrm{Pe}^2}$ and is thus controlled by a combination of propagation and diffusion, and the assumption of a small front width is justified if $\sqrt{\mathcal{D}} \ll 1$ and Pe is not too large.

d. Front motion. The time scale of advection by tissue flow is ν/T_0, i.e. the time scale of mechanical tissue contraction. Experiments suggest that typical contraction rates are on the order of 0.2 hr^{-1}, corresponding to a tissue motion time scale of about 5 hours, which is much longer than the biological regulation which is supposed to occur on the scale of minutes or a few dozen minutes. As a result, the model generically predicts the formation of a bi-contractile state with moving fronts that gradually converge towards steady state. The velocity of such a front v may be approximated by considering a solution $T_a(s_f = s - vt)$ to Eq. (12) and integrating it across a front after multiplication by $\partial_s T_a$. From Eq. (21) we have $\int (\partial_s T_a)^2 \, \mathrm{d}s \approx \zeta^2/\sqrt{\mathcal{D}}\sqrt{1+\mathrm{Pe}^2}$ and so in the limit of sensitive regulation $\beta \gg 1$ we find

$$v \approx \mathcal{P}u(s_f) + \frac{2\sqrt{\mathcal{D}}\sqrt{1+\mathrm{Pe}^2}}{\zeta}\left(T - 1 - \frac{\alpha}{\beta}\right). \tag{23}$$

From this we learn that the velocity at which the fronts move relative to the tissue is controlled predominantly by the tension in the margin, with lower tension promoting the expansion of the contracting domain. In steady state $v = 0$ and refined approximations for the tension T, contractile domain size ρ, peak tissue velocity u_0 and Péclet number Pe are ultimately found to be

$$T \approx 1 + \frac{\alpha}{\beta} - \zeta\frac{\mathrm{Pe}}{\sqrt{1+\mathrm{Pe}^2}}, \tag{24a}$$

$$\rho \approx \frac{1}{2}\left(1 + \frac{\alpha}{\beta\zeta} - \frac{\mathrm{Pe}}{\sqrt{1+\mathrm{Pe}^2}} + 8\sqrt{\mathcal{D}}\mathrm{Pe}\right), \tag{24b}$$

$$u_0 \approx \frac{\zeta}{4}\left(1 - \frac{\alpha^2}{\beta^2\zeta^2}\right), \tag{24c}$$

$$\mathrm{Pe} \approx \frac{\zeta\mathcal{P}}{8\sqrt{\mathcal{D}}}\left(1 - \frac{\alpha^2}{\beta^2\zeta^2}\right). \tag{24d}$$

Comparing with the simulation results in Supp. Fig. 2, we see that these estimates are rather accurate, even for a large value of $\mathcal{P}$. The error in the estimate for T is 2.2%, and in the estimate for ρ it is 11%. Conversely, taking the values of T and $u(s_f)$ from the simulation we find that Eq. (23) predicts an error $v/u(s_f) \approx 7\%$.

In conclusion, convergence towards a steady pattern in the model results from the motion of fronts between the contracting and stretched domains, which itself depends on the balance between advection, which tends to narrow contracting domains, and a 'restoring force' that tends to bring the proportions of the domains to a point where tension along the margin matches a 'homeostatic tension' $1 + \alpha/\beta$. The strength of this restoring force depends on the effective diffusivity, i.e. on the non-local activation of contractility, and on the rate of regulation, which must both be large enough to maintain a stable contractile domain.

e. Rescaling after ablation. The analysis so far has assumed a strictly positive tension, as in the uniform steady state. This explains the regulation of contractile and extensile regions in a periodic domain, or a domain with fixed end points as in the case of reattachment after ablation. In particular, since the ratio of contractile and extensile domains ρ depends only on α and not on the length of the margin, the model predicts that the contractile region rescales after ablation if the ends reattach (provided the instability criterion Eq. (16) is still satisfied, but this also depends only weakly on the size of the domain through the non-dimensional diffusivity $\mathcal{D}$, and so is in general still going to be satisfied for reasons outlined previously). In contrast, when the ends do not reattach, the model predicts very different behaviour. In this case, the overall tension is rapidly reduced to $T = 0$. As illustrated in Supp. Fig. 3, this tension generically lies outside of the admissible window for bi-contractility and there is only a single stable steady state, corresponding to a uniform contraction with

$$\dot{\epsilon}_{\mathrm{retr.}} \approx -1 - \zeta. \tag{25}$$

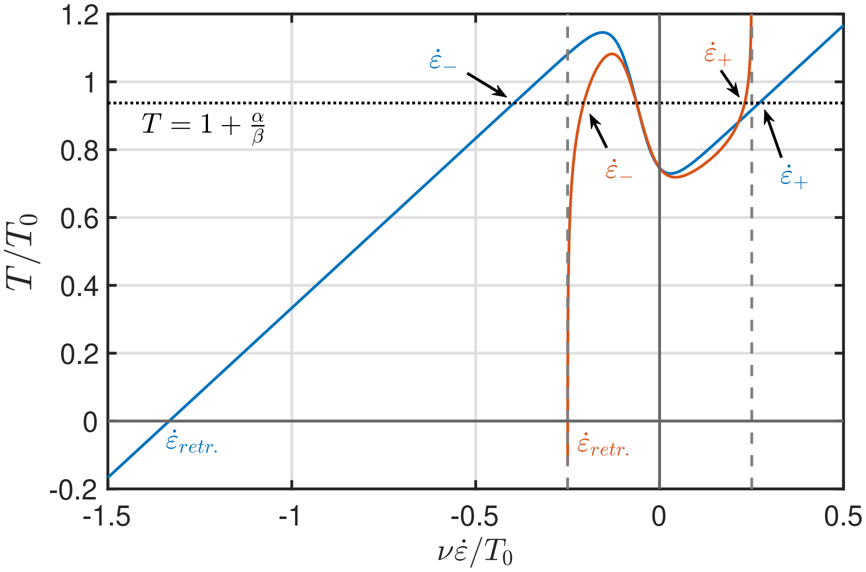

Supplementary Figure 3: Illustrative steady state total tension for the minimal model (blue) and the full model model with non-linear walking kernel (orange). For the minimal model, the steady state tension can be calculated approximately (ignoring corrections from advection and diffusion), predicting the coexistence of extensile $\dot{\epsilon}_+ > 0$ and contractile $\dot{\epsilon}_- < 0$ regions. In the absence of tension, as in the case of ablation, the model predicts a uniform negative contraction rate $\dot{\epsilon}_{\text{retr.}}$. While the minimal model predicts a large ratio $\dot{\epsilon}_{\text{retr.}}/\dot{\epsilon}_-$, this is rectified through the introduction of the non-linear walking kernel in the full model. Simulation parameters as in Table 1: $\alpha = -1$, $\beta = 16$, $\zeta = 0.33$, $\lambda = 4$.

While this is in agreement with experimental observations qualitatively, we note that the ratio between the release contraction rate and the contraction rate in steady state is predicted to be rather large,

$$\frac{\dot{\epsilon}_{\text{retr.}}}{\dot{\epsilon}_-} \approx \frac{1 + \zeta^{-1}}{1 - \frac{\alpha}{\beta\zeta}} \gg 1, \tag{26}$$

for reasonable values of the parameters. This contrast with experimental observations is ultimately due to the limitations of the minimal model. This is rectified by means of a non-linear description of the contractility dynamics in the full model in section I C and also illustrated in Supp. Fig. 3 (orange curve).

C. Non-linear 2D model with surrounding tissue

1. Derivation

a. General case. While the 1D model serves well in providing a qualitative understanding of the self-regulation, it neither provides for a complete description of tissue flows in the embryo and their feedback onto the regulation, nor does it accurately predict the retraction speed upon ablation. In order to address these shortcomings, we now introduce a full two-dimensional model of the regulation dynamics in the embryo that differs in two key aspects: first, the regulation dynamics of the margin are fully coupled to the motion of the underlying tissue, and second the generation of active stresses in the margin is modelled explicitly in terms of actomyosin cables with varying density. By modelling the tissue motion and growth we are able to apply the model directly to different embryonic geometries and boundary conditions, such as ablated posterior and anterior halves, and numerically predict the location and size of contractile regions, as well as the morphology of the embryo. By allowing for a non-linear relation between the load borne by supra-cellular cables and the rate at which they contract, the extended model is able to capture the controlled contraction speed after ablation. It also takes into account explicitly the three different time scales of the dynamics, namely those for biological regulation, build-up of tension in the margin, and advection speed of the tissue, as opposed to the minimal model which focused on the biological regulation only.

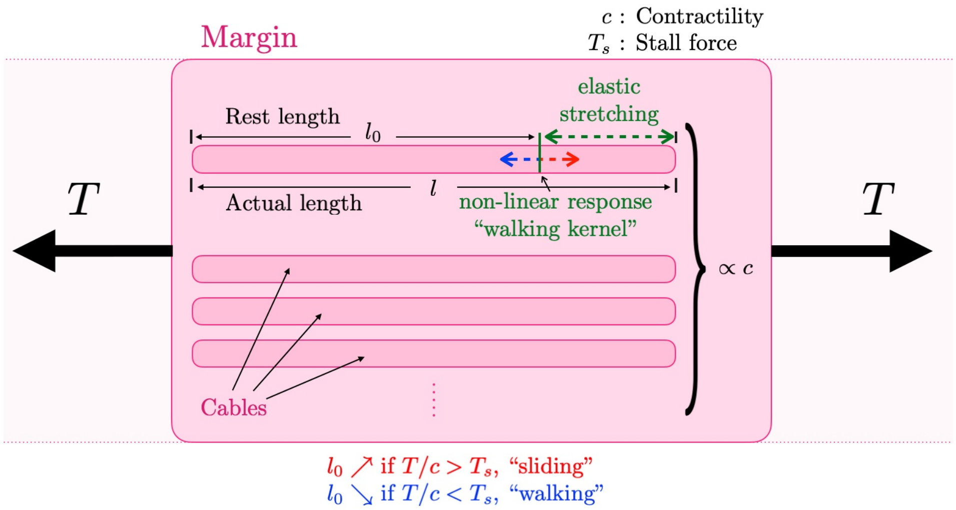

Supplementary Figure 4: Illustration of the non-linear walking kernel in the 2D model. Elastic actomyosin cables are stretched elastically from their rest length l_0, while the rest length itself responds to mechanical stresses in a non-linear fashion. If the tension is below the stall force of the myosin motors multiplied by the density of actomyosin, the cables contract, otherwise they expand. The non-linear nature of the response ensures that the extension/contraction rate never exceeds a given bound.

Departing from the minimal model that represents margin contractility in terms of a time-dependent active tension profile, we now explicitly consider the actomyosin cables that make up the margin as active, viscoelastic elements, with a non-linear behaviour that includes a saturation of the contraction rate in the case of very high or low tension. Specifically, the rest length $l_0(s,t)$ of a margin element responds to a given tension $T(s,t)$ as

$$\frac{1}{l_0}\frac{\partial l_0}{\partial t} = \lambda \dot{\epsilon}_0 W\left(\frac{T}{cT_s};\lambda\right) + \frac{\gamma}{2}, \quad W(x;\lambda) = \lambda^{-1}\tanh\lambda\,(x-1)\,, \tag{27}$$

where define the walking kernel W in reference to [24], and the term $\gamma/2$ is included to allow for the shrinking of cables through cell ingression at the primitive streak (absent a detailed characterization of the effect of cell ingression on supracellular cables, areal contraction is equated with isotropic contraction at a rate $\gamma/2$ in each direction). Here we introduce $c(s,t)$ as a variable for contractility, which can be understood to represent the local density of active myosin or supracellular cables, and T_s a stall force per unit contractility at which junctions transition from contraction to yielding. As is illustrated in Supp. Fig. 5, the kernel W is constructed in such a fashion as to allow for viscous sliding when the load T is close to the equilibrium value cT_s, but saturates to a maximal contraction rate, $\dot{\epsilon}_0 \tanh\lambda$, when the tension is released, $T = 0$. Here λ regulates the degree of non-linearity and for $\lambda > 1$ the maximal contraction rate is very close to $\dot{\epsilon}_0$. The kernel captures the idea that in the absence of tension myosin walkers contract with a fixed rate $\dot{\epsilon}_0$ that is independent of the number of walkers present. If the load is increased, the walkers need to overcome resistance to motion and stall at a load of T_s per unit concentration. When the load is increased further, the actomyosin cables begin to yield and eventually reach a maximal extension rate. An illustration of the walking kernel is provided in Supp. Fig. 4, while the behaviour of the function W is plotted in Supp. Fig. 5.

Having described the time evolution of the rest length of individual margin segments, we write an equation for the evolution of elastic tension along the margin as

$$\frac{\partial T}{\partial t} + u\frac{\partial T}{\partial s} = E\left[\dot{\epsilon} - \frac{\gamma}{2} - \frac{\dot{l}_0}{l_0}\right], \tag{28}$$

where E is the elastic modulus of the actomyosin cables. This equation captures the idea that the cables respond elastically when stretched from their rest length, but that elastic stresses can be released through a dynamic adjustment of this rest length under load. Combining these two elements effectively yields a non-linear viscoelastic Maxwell-like behaviour. We proceed by recasting our model equation for the regulation of active tension in the minimal model,

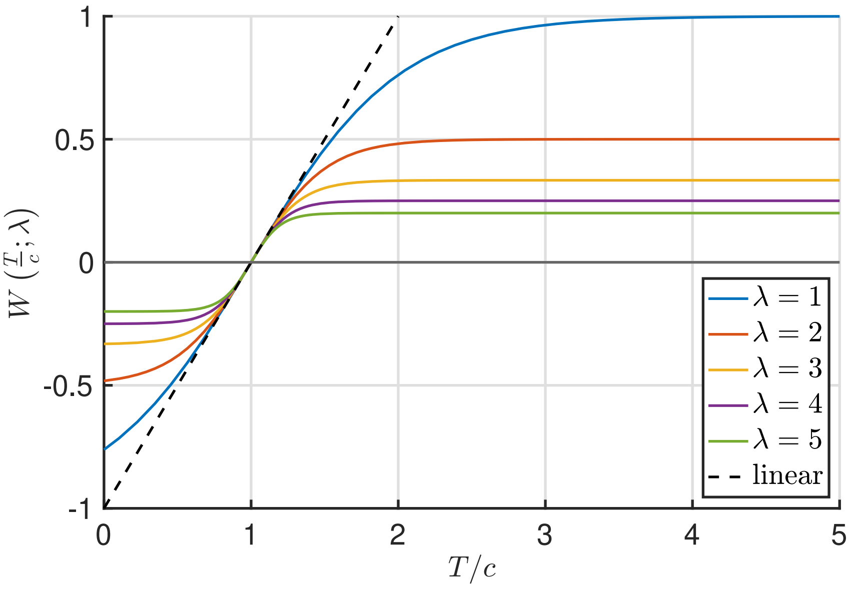

Supplementary Figure 5: Illustration of the walking kernel $W(T/c;\lambda)$ for different values of λ and comparison with the linearised kernel Eq. (38) for the case $\zeta \ll 1$. The parameter λ controls the degree of non-linearity. In the absence of tension, $T = 0$, the walking kernel is bounded by $-1/\lambda$, ensuring that contraction rates are not too big when tension is released.

Eq. (7), as an equation for the contractility c or density of these active elements,

$$\frac{\partial c}{\partial t} + u\frac{\partial c}{\partial s} = \frac{1}{\tau}\left[c_0 + \Delta c \tanh\left(\alpha - \frac{\beta\dot{\epsilon}}{\lambda\dot{\epsilon}_0}\right) - c\right] + D\frac{\partial^2 c}{\partial s^2}. \tag{29}$$

Here we introduce a reference contractility c_0 and a contractility amplitude Δc. To incorporate the coupling between the margin and the surrounding tissue, we follow [6] and model the embryonic disk as a two-dimensional Stokes fluid with a prescribed divergence $\gamma(\boldsymbol{x},t)$ and active stresses $\boldsymbol{\sigma}_a(\boldsymbol{x},t)$,

$$-\boldsymbol{\nabla} p + \mu\nabla^2\boldsymbol{u} + \boldsymbol{\nabla}\cdot\boldsymbol{\sigma}_a = \boldsymbol{0}, \quad \boldsymbol{\nabla}\cdot\boldsymbol{u} = \gamma, \tag{30}$$

where μ is the dynamic viscosity of the tissue, $\boldsymbol{u}(\boldsymbol{x},t)$ the 2D flow velocity and $p(\boldsymbol{x},t)$ the pressure. Following [6], we define the area changes $\gamma(\boldsymbol{x},t)$ analytically using five modes that approximate the average growth of the embryo in reference to the initial geometry. We summarise the form of these in Table 2 and refer the reader to section S7.7 in the SI to [6] for details. We note that the dominant contributions to area changes (modes 3 and 4 in Table 2) represent a radially symmetric expansion of the extra-embryonic tissue, with no incidence on motion along the margin. Treating the contractile cables within the margin as viscoelastic but the surrounding tissue as a fluid greatly simplifies the analysis of motion driven by the margin, and is also justified by the observation that tissue outside the margin more readily dissipates tension, whereas larger elastic strains build up in the margin, as revealed by laser ablation experiments [6].

The margin is immersed in the tissue and the locus of its centreline is described by the position vector $\boldsymbol{r}(s,t)$; by definition of s as arc length the tangent $\boldsymbol{t} := \partial\boldsymbol{r}/\partial s$ is hence a unit vector. The active stress in the tissue $\boldsymbol{\sigma}_a(\boldsymbol{x},t)$ is a second-rank tensor and instantaneously given by

$$\boldsymbol{\sigma}_a(\boldsymbol{x},t) = \frac{e^{-y^2/2d^2}}{\sqrt{2\pi d^2}}T\left(s\left(\boldsymbol{x}\right)\right)\boldsymbol{t}\otimes\boldsymbol{t} \tag{31}$$

where $s(\boldsymbol{x})$ corresponds to the point on the margin closest to $\boldsymbol{x}$, $y = |\boldsymbol{x} - \boldsymbol{r}(s(\boldsymbol{x}))|$ and d a parameter that provides a scale for the margin width. This assumes that regulation along the margin occurs without any cross-sectional variation, and hence remain a one-dimensional problem described by Eqs. (28) and (29) with $u = \boldsymbol{t}\cdot\boldsymbol{u}(\boldsymbol{r})$ and $\dot{\epsilon} = \boldsymbol{t}\cdot\boldsymbol{\nabla}\boldsymbol{u}(\boldsymbol{r})\cdot\boldsymbol{t}$. At the same time, the stress profile across the margin is described by a Gaussian to reflect the fact that it is slightly fuzzy with multiple actomyosin cables spread in parallel. The normalization is chosen to ensure that the net stress

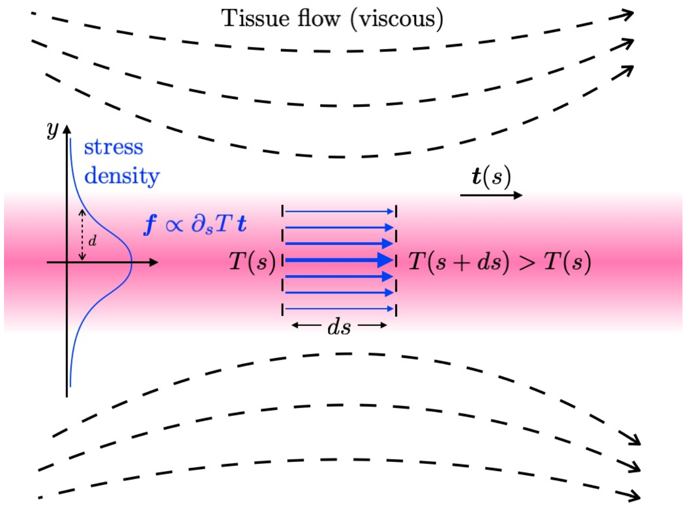

Supplementary Figure 6: Illustration of the coupling between margin and surrounding tissue in the extended model. Differences in tension along the margin result in an apparent force density $\nabla \cdot \boldsymbol{\sigma}_a = \boldsymbol{f}$ on the surrounding tissue, modelled as a 2D viscous fluid. These stresses are parallel to the margin and distributed in a Gaussian profile in the orthogonal direction. The position of the margin centreline and its tangent are themselves not fixed but dynamically advected with the flow.

applied to the tissue is independent of the width parameter d. In the limit where the margin width $d \to 0$, the force distribution becomes singular on $\boldsymbol{r}(s)$. A sketch of the interaction between margin and surrounding tissue is given in Supp. Fig. 6.

In summary, the regulation dynamics are fully described by the coupled system Eqs. (28)-(31). Conceptually, the instantaneous distribution of stresses in the margin determines the tissue flow through Eq. (30), which in turn informs the evolution of biological contractility and mechanical stresses in Eqs. (28)-(29) and the advection of margin elements with the tissue. This extended 2D model exhibits a number of qualitative differences compared to the 1D minimal model, the most significant of which are a bound on the contraction rate and the possibility (indeed, the requirement) of a non-uniform tension profile T. In the following sections, we analyze these differences in detail and explain why they are necessary to accurately describe the biological experiments.

b. Analytical simplification in a straight periodic geometry. The theoretical analysis of the 2D model is greatly simplified when the margin is assumed straight, with $\boldsymbol{t}$ constant, since this allows for an explicit solution of the Stokes equations Eq. (30). In this section and the remainder of section IC we also ignore the effect of area changes, $\gamma = 0$, since these do not directly influence the tension profile along the margin (up until the onset of cell ingression at the primitive streak, at least).

We imagine a Cartesian coordinate system (x, y) with the margin centreline on the x-axis and period L. The tissue velocity $\boldsymbol{u}$ decays to zero as $y \to \pm\infty$, far away from the margin. Ignoring the effect of tissue growth, the flow field is incompressible and hence can be described by a scalar stream function $\psi(x, y)$ that satisfies the biharmonic equation $\nabla^4 \psi = 0$. The velocity field may then be recovered as $\boldsymbol{u} = (u_x, u_y) = (\partial_y \psi, -\partial_x \psi)$.

Because of the periodicity of the margin, the tension may be written in terms of Fourier modes $T(x) = T_n e^{ik_n x}$ where $k_n = 2\pi n/L$ and n is a positive integer labelling the mode. Using an ansatz with similar periodicity for the stream function, $\psi = \psi_n(y) e^{ik_n x}$, the Stokes equations Eq. (30) simplify to an ordinary differential equation for ψ_n,

$$\mu \left(-k_n^2 + \frac{\partial^2}{\partial y^2} \right)^2 \psi_n = \frac{ik_n}{\sqrt{2\pi}} \frac{y}{d^3} e^{-y^2/2d^2} T_n, \tag{32}$$

which can be solved to find ψ_n explicitly in terms of exponentials and the error function. Requiring boundedness as $y \to \pm\infty$ and up-down symmetry is sufficient to ensure uniqueness of the solution. From this, it may be shown that the nth mode of the horizontal extension rate, defined as $\dot{\epsilon}_n = ik_n \partial\psi_n/\partial y$ at $y = 0$, is related to the nth tension mode

T_n by

$$\dot{\epsilon}_n = -\frac{1}{\mu d} f\left(k_n d\right) T_n, \tag{33}$$

where the transfer function f is given by

$$f(x) = \frac{x}{4}\left(-x\sqrt{\frac{2}{\pi}} + (1+x^2)e^{x^2/2}\,\mathrm{erfc}\left(\frac{x}{\sqrt{2}}\right)\right) = \frac{x}{4} + \mathcal{O}\left(x^2\right). \tag{34}$$

The response function f is strictly positive, grows linearly for long wavelengths ($f \sim k_n d$ as $k_n d \to 0$) and decays quadratically as $k_n d \to \infty$. The decay for large $k_n d$ ensures mechanical robustness of the tissue flow to small scale perturbations. It is due to the non-local entrainment of the tissue by the Gaussian distribution of actomyosin cables, and disappears for wavelengths significantly longer than the margin cross-sectional width, $k_n d \gg 1$, this effect disappears and we approximate $f \sim x/4$ to simplify the theoretical analysis.

Further insight may be gained by scaling the model equations to obtain all dimensionless parameters. To this end, we scale contractility by c_0, tension by $c_0 T_s$, lengths by L, time by the regulation time scale τ, while the extension rate is scaled by $\lambda\dot{\epsilon}_0$ and velocity by $L\lambda\dot{\epsilon}_0$ for reasons that will become clear shortly. This leads to the three scaled equations

$$\boxed{\begin{aligned}
\frac{\partial T}{\partial t} + \mathcal{P}\,u\frac{\partial T}{\partial s} &= \mathcal{E}\left[\dot{\epsilon} - W\left(\frac{T}{c};\lambda\right)\right], && \text{(35a)}\\
\frac{\partial c}{\partial t} + \mathcal{P}\,u\frac{\partial c}{\partial s} &= 1 + \zeta\tanh\left(\alpha - \beta\dot{\epsilon}\right) - c + \mathcal{D}\frac{\partial^2 c}{\partial s^2}, && \text{(35b)}\\
\dot{\epsilon}_n &= -\frac{n\pi}{2\mathcal{T}}T_n, && \text{(35c)}
\end{aligned}}$$

where we define

$$\mathcal{P} = \tau\lambda\dot{\epsilon}_0, \quad \mathcal{E} = \frac{E\tau\lambda\dot{\epsilon}_0}{c_0 T_s}, \quad \mathcal{T} = \frac{\mu L\lambda\dot{\epsilon}_0}{c_0 T_s}, \quad \mathcal{D} = \frac{D\tau}{L^2}, \quad \zeta = \frac{\Delta c}{c_0}, \tag{36}$$

which together with α, β and λ adds up to **eight model parameters**, compared with five in the 1D model. An additional ninth parameter, $\delta = \frac{d}{L}$, for the width of the margin is retained in simulations but omitted in the analysis since it is fixed to a known value by the geometry of the embryo and small enough to have a negligible effect.

While some of these dimensionless parameters may appear counter-intuitive at first, their meaning becomes clearer when we define $\nu = c_0 T_s/\lambda\dot{\epsilon}_0$ and $T_0 = c_0 T_s$ in analogy with the viscous internal dissipation of the margin in the minimal model. In this case the coefficients may be rewritten as

$$\mathcal{P} = \frac{T_0\tau}{\nu}, \quad \mathcal{E} = \frac{E\tau}{\nu}, \quad \mathcal{T} = \frac{\mu L}{\nu}, \tag{37}$$

which not only makes explicit their connection with the minimal model, but also gives a physical meaning to $\mathcal{E}$ as the ratio of the regulation time to the time for tension build-up, and $\mathcal{T}$ as the ratio of viscous dissipation in the tissue to that in the margin. As before, $\mathcal{P}$ quantifies the importance of advection on the time scale of regulation. Finally, in steady state the (dimensionless) contraction rate $\dot{\epsilon}$ is bounded by λ^{-1}, which makes it possible to interpret λ as a measure of the non-linearity of the walking kernel.

2. The 1D model as a limiting case

It is now straightforward to recover the minimal model from the scaled equations of the complete model, Eqs. (35a)-(35c). Specifically, it emerges in the case when the actomyosin cables of the margin are very stiff compared to the viscous forces that are generated internally by the sliding myosin crawlers, and if both of them are much bigger than the viscous dissipation in the surrounding tissue. Mathematically, this corresponds to the condition $E\tau \gg \nu \gg \mu L$, or $\mathcal{E} \gg 1 \gg \mathcal{T}$ in terms of the dimensionless parameters. In addition, it is necessary consider a margin of vanishing cross-section $\delta \to 1$, consistent with the picture of a 1D line in the context of the minimal model.

We demonstrate this by considering a small perturbation from the equilibrium solution of Eqs. (35a)-(35c). In the absence of any motion, $u = \dot{\epsilon} = 0$ and the tension and contractility have the identical uniform value $T = c =$

$1 + \zeta \tanh\alpha$. Considering the ansatz $T = 1 + \zeta \tanh\alpha + T'$ and similar for c, we find that the walking kernel W may be linearised as

$$W\left(\frac{T}{c};\lambda\right) \approx T' - c' = T - c. \tag{38}$$

The condition $\mathcal{E} \gg 1$ applied to Eq. (35a) implies that the right-hand side must balance itself, and so

$$0 = \dot{\epsilon} - T + c, \tag{39}$$

or, reinserting dimensions,

$$T = \frac{c_0 T_s}{\lambda \dot{\epsilon}_0}\dot{\epsilon} + T_s c, \tag{40}$$

which is equivalent to Eq. (8) if we identify $T_a = T_s c$. Hence we recover that the active tension in the minimal model is equivalent to the contractility in the linearised 2D model. At the same time, this provides a justification for the definition of ν in the previous section as an effective linear viscosity that emerges through the sliding of the myosin motors. It then follows that Eq. (35b) corresponds precisely to Eq. (7) up to a constant of proportionality. Finally, the condition $\mathcal{T} \ll 1$ ensures that an $\mathcal{O}(1)$ contraction or extension is compatible with a small tension perturbation as assumed initially. The limitation of the argument by the approximation Eq. (38) restricting the margin tension T to small perturbations from equilibrium highlights clearly why 1D the minimal model cannot capture the full behaviour of the 2D non-linear model, when tension is suddenly released.

3. New predictions

a. Most unstable wavelength. We revisit the stability analysis of section I B 2 a to include the effect of the surrounding tissue. Within the framework of a linear stability analysis, the non-linearity of the walking kernel does not enter. We consider the dimensionless system of equations. We examine the stability of the uniform state $T = c = 1 + \zeta \tanh\alpha$, $\dot{\epsilon} = 0$ by assuming a perturbation of the nth mode, proportional to $\exp(i2\pi ns + \sigma_n t)$. Linearising the system Eqs. (35a)-(35c) then sets a quadratic condition on the growth rate σ_n,

$$\left(\sigma_n + 1 + 4\pi^2 n^2 \mathcal{D}\right)\left(\sigma_n + \mathcal{E}\left(1 + \frac{n\pi}{2\mathcal{T}}\right)\right) = \frac{n\pi}{2\mathcal{T}}\mathcal{E}\beta\zeta\,\mathrm{sech}^2\,\alpha. \tag{41}$$

This enables a positive growth rate (and thus instability) if and only if

$$\beta\zeta\,\mathrm{sech}^2\,\alpha > \left(1 + \frac{2\mathcal{T}}{n\pi}\right)\left(1 + 4\pi^2 n^2 \mathcal{D}\right). \tag{42}$$

As in the case of the minimal model, which we recover in the limit of vanishing tissue viscosity $\mathcal{T}$, Eq. (16), this essentially amounts to a condition on the regulative sensitivity β to be sufficiently large, while the spatial extent of actomyosin cables both tangentially (through $\mathcal{D}$) and orthogonally (through δ, c.f. the asymptotic behavior of f discussed in section I C 1 b) to the margin ensures that short wavelength instabilities, as for example due to noise, are suppressed. In contrast however, the fundamental mode $n = 1$ is no longer guaranteed to be the most unstable, instead there is a competition of hydrodynamic and regulative effects. To leading order in the stiffness $\mathcal{E}$ and tissue viscosity $\mathcal{T}$, the most unstable mode is found to be

$$\boxed{n \approx \frac{\beta\,\mathrm{sech}^2\,\alpha\mathcal{T}}{4\pi^3\mathcal{D}}}. \tag{43}$$

This shows that an increase in tissue viscosity favours the formation of ectopic contractions.

b. Effect of the surrounding tissue on margin regulation. In order to better understand the effect of the inclusion of tissue motion through the parameter $\mathcal{T}$, it is instructive to consider the mode relationships between contractility, tension and extension rate. For a step-like profile of the contractility c we have the scaling $c_n \sim \zeta/n$ by the properties of Fourier series. Assuming a small perturbation, we can substitute Eq. (35c) into Eq. (39) to obtain an approximate scaling for T_n. Re-substituting into Eq. (35c) yields a scaling for $\dot{\epsilon}_n$, from which a scaling for u_n may be obtained following the rules for the integration of a Fourier series. In summary,

$$c_n \sim \frac{\zeta}{n}, \quad T_n \sim \frac{\zeta\mathcal{T}}{n(\mathcal{T}+n)}, \quad \dot{\epsilon}_n \sim -\frac{\zeta}{\mathcal{T}+n}, \quad u_n \sim -\frac{\zeta}{n(\mathcal{T}+n)}, \tag{44}$$

for $n > 1$. From this we see again that in the case of vanishing tissue viscosity $\mathcal{T}$, there is no variation in tension. However, it also becomes evident that the inclusion of the tissue regularizes the contraction and velocity profiles $\dot{\epsilon}$ and u in response to the contractility c by acting as a high-pass filter. Thus the effect of the tissue manifests in a reduction of the magnitude of the extension rate in the centre of extensile regions, as well as the generation of a tension peak in contractile regions.

c. Stability of the stretched anterior at long times Physically, the 'drag force' of the surrounding tissue screens the propagation of tension along the margin. If transmission along the margin is too strongly hindered, and the stretching imposed in extensile regions too strongly reduced, these regions may become unstable toward the formation of an ectopic contraction. This is reflected in the linear stability analysis carried out above, which shows that the fundamental mode $n = 1$ may not be the most unstable if the tissue viscosity $\mathcal{T}$ is large, and is exacerbated in the non-linear regime where advection further shrinks contractile regions and extends extensile regions. We may illustrate this by considering a generalization of Eq. (42) to a perturbation about a state with a uniform extension rate $\dot{\epsilon}_+$ (as is approximately the case in the anterior). In that case an instability occurs if

$$\beta\zeta \operatorname{sech}^2(\alpha - \beta\dot{\epsilon}_+) > \left(1 + \frac{\mathcal{T}\delta}{f(2\pi n\delta)}\right)\left(1 + 4\pi^2 n^2 \mathcal{D}\right), \tag{45}$$

For negative α and positive β (as considered here), the left hand side increases as $\dot{\epsilon}_+$ increases towards zero, and so may eventually grow sufficiently large for an ectopic instability to occur. For the parameters chosen in our numerical simulations, listed in Table 1, this does not occur, but we find numerically that ectopic instabilities do occur for sufficiently large values of the tissue viscosity $\mathcal{T}$ and the propagation number $\mathcal{P}$. In conclusion, our model predicts that the robust formation of a single contraction in wild type embryos places constraints on the ratio of viscosities between the margin and the surrounding tissue.

d. Front motion in the non-linear model. In a similar fashion to the 1D model, it is possible to gain insight into the motion of fronts in the extended non-linear model. Assuming that advection of tension (unlike advection of contractility) plays a limited role, as supported by our numerical simulations, we have $\dot{\epsilon} \approx W(T/c; \lambda)$. If we approximate the tension, like the velocity, as uniform across the front, $T \approx T(s_f)$, we can rewrite Eq. (35b) as

$$\frac{\partial c}{\partial t} + \mathcal{P}\, u \frac{\partial c}{\partial s} = 1 + \zeta \tanh\left(\alpha - \beta W\left(\frac{T(s_f)}{c}; \lambda\right)\right) - c + \mathcal{D}\frac{\partial^2 c}{\partial s^2} \tag{46}$$

In the limit where $\beta/\lambda \gg 1$, we can make the further approximation

$$\tanh\left(\alpha - \beta W\left(\frac{T(s_f)}{c}; \lambda\right)\right) \approx \tanh\left(\alpha - \frac{\beta T(s_f)}{c}\right) \tag{47}$$

Thus, we recover an equation that is very similar in form to the equation for the time evolution of active tension in the minimal model Eq. (12). Following the same steps as in that case, we find that the same equation relating the velocity of the front to the local tissue velocity and tension, Eq. (23), applies. In order words, whereas a more extensive analysis of the full nonlinear model may be complicated by the nonlinear equation of motion relating tension, velocity, and contractility, its behavior can be understood in the same way, as resulting from a balance between advection and tension homeostasis.

II. SUPPLEMENTARY METHODS

A. Numerical simulations – Implementation

In order to compare our theoretical predictions with experiments, we implemented the model described in section I C numerically using the finite element package FEniCS 2019.1.0 [34, 35]. The numerical routine is iterative, solving the Stokes equations Eq. (30) using finite elements to obtain the instantaneous flow field $\boldsymbol{u}$ from the stress distribution $\boldsymbol{\sigma}_a$ in alternation with explicit Euler integration of the system Eqs. (28)-(29) to update the stresses. The margin is modeled as a chain of N straight elements, the end points of which are advected with the flow field. N is chosen in order to generate margin segments with a length typical for the mesh size. For a mesh resolution of 0.1 mm, this yields $N = 29$ for an embryo half and $N = 58$ for a complete embryo. The contraction rates along each segment are calculated directly from the kinematics of the endpoints. The tension and contractility changes on the margin

elements are calculated locally in a Lagrangian fashion, hence the advective terms of Eqs. (28)-(29) (which are stated in the Eulerian picture) are not included explicitly.

The mesh is auto-generated by the finite element package with a homogeneous density corresponding to at least twice the resolution of the margin segments. The mesh is created in such a way that ensures that each margin segment coincides with an edge of the mesh in order to maximise numerical accuracy. The finite elements themselves are Lagrangian third-order for the velocity and second-order for the pressure (i.e. second-order Taylor-Hood elements), and the mesh is advected with the flow. In order to maintain a homogeneous mesh, the domain and margin are remeshed at regular intervals. Furthermore, the tension distribution T is interpolated linearly between the midpoints of the margin segments in order to smooth out the stress distribution $\boldsymbol{\sigma}_a$ acting on the fluid.

In order to account for tissue growth and for cell ingression at the primitive streak, we also account for area changes by including a source term γ in the Stokes equations (a term in γ also appears in Eq. (28) to allow for the shrinking of cables through cell ingression). These area changes are prescribed in a fashion that closely approximates the experimentally observed behavior [6], and can be decomposed into five different modes, detailed in Table 2 (see S7.7 in the SI to [6] for details). These area changes are applied in a Lagrangian fashion, with each finite element having an appropriate value of γ attributed at the initialization stage which it maintains over the course of the simulation. Upon remeshing, the new divergence values are obtained by interpolation. As noted under Methods, the mode corresponding to cell ingression at the primitive streak is included when modelling the intact epiblast, as well as the asymmetric perturbations in Extended Data Fig. 8, but omitted for posterior halves (main Fig. 4) and experiments with a full obstacle (main Fig. 5), since we do not wish to explicitly model the redirection of ingression accompanying the redirection of embryo formation.

The boundary conditions of the finite element routine are implemented slightly differently depending on the experiments being modeled, as we explain below.

B. Numerical simulations – Choice of parameter values and boundary conditions

A list of parameters used for the simulations is given in Table 1. For all of the them, we can derive quantitative, or at least qualitative constraints, from our experimental observations ([6] and this study). When constraints are qualitative, we choose parameter values such that the relevant dimensionless quantities satisfy the constraints without excessively deviating from unity, a natural prior that disfavors biologically unreasonable values. Several parameters are required to be sufficiently large to yield sufficiently strong nonlinearities in the model. For instance, the sensitivity β must be sufficiently large for spontaneous symmetry breaking, as observed in anterior epiblast halves; the value $\beta = 16$ is chosen to be about twice (i.e., larger but not vastly larger than) the threshold from Eq. (2) given the values of α and ζ. Likewise, the value of λ is chosen to be sufficiently large for the contraction rate in the posterior not to be limited by tension, and the tissue viscosity $\mathcal{T}$ sufficiently small for tension to propagate and inhibition to be effective in the anterior, as analysed in section I C 3 c.

Among the parameters that are quantitatively constrained, the margin length and width are taken from [6]; the length was directly measured, while the width was obtained by fitting a model to the experimentally observed tissue flows, and subsequently confirmed based on the observed distribution of phosphorylated myosin in fixed embryos. Likewise, the parameters in Table 2 describing area changes (which have no incidence on regulation in the model) are taken from [6] and were fit to experiments. The stiffness of the margin (relative to the magnitude of active tensions) is chosen in accordance with the observed elastic strains at the margin ($\sim 10\%$, cf. [6]), which yields $E \sim 10$. Ablation experiments revealing tensions in the anterior that are comparable to tensions in the posterior suggest that ζ must smaller than one (absent a more precise measurement, we chose $\zeta = 1/3$ such that the tension in the anterior is half the tension in the posterior). Based on the rapid redirection of tissue motion in our experiments, the regulation time scale τ is chosen to be under an hour (an order of magnitude that is compatible with the turnover of supracellular cables at the margin, as observed in [6]; the small regulation time scale $\tau = 1/2$ also implies a value of the propagation parameter $\mathcal{P}$ below one, cf. Eq. (36)). The effective myosin diffusivity D is then chosen to match the estimated width of the fronts, which based on both velocity profiles and the observed length of supracellular cables is commensurate with the width of the margin, in the hundreds of microns.

With the above constraints on parameters satisfied, the essential qualitative features of the model, as analyzed in the previous sections, are fixed. For instance, the velocity along the margin exhibits an approximately triangular profile, and is predicted to rescale according to the margin size. This leaves as quantitative features to be adjusted the extent ρ of the contractile domain occupied by the contracting domain, and the strain rate within that domain. The parameter α, which controls the extent of the contractile domain, was chosen such that 2D simulations match the observed extend of the contractile domain (just under 1mm, cf. Fig. 1c; values $\sim 1/3$ - below a half - imply $\alpha < 0$), and the maximal contraction rate $\dot{\epsilon}_0$ was chosen to match the 95% contraction of the posterior margin in a

reference period of 8h [6].

a. Intact embryo (main Fig. 1, Supplementary Video 1) In this case the margin is fully immersed within the domain and is not subject to a boundary condition. The initial shape of the embryo is circular with radius 1.81mm, while the initial locus of of the margin is circular with radius 0.92mm and offset toward the posterior by a distance of 0.106mm. At the outer edge of the embryo a no-slip condition is applied, and it is assumed that the embryo remains in circular shape with a radial boundary velocity $u_n(t)$. This velocity is calculated at each time step from the area changes γ using the divergence theorem. The embryo is initialized with uniform tension equal to the homogeneous equilibrium value, $T = 1 + \zeta \tanh \alpha$, and a sinusoidal bias in contractility c in the anterior, with amplitude ζ, $c = 1 + \zeta \cos \theta$ where θ is the polar angle measured from the posterior pole.

b. Posterior and anterior halves (main Figs. 3 and 4, Supplementary Videos 3 and 5) In this case the boundary of the embryonic half is set by a linear cut orthogonal to the anterior-posterior (A-P) axis and through the center of the embryo proper. On the cut a no-slip condition is applied in the case of reattachment, and a no stress condition is applied in the case of no reattachment. The outer boundary moves with a velocity $\boldsymbol{u}_n(t) \propto \boldsymbol{n}$ that is determined as in the intact case from the total area changes. The margin has two end points which are situated on and advected with the cut, where a no flux boundary condition is applied to the contractility c. The no-slip condition for a reattached border is enforced through a penalty term to the finite-element energy functional of the form $||\boldsymbol{u}_{\text{boundary}} - \boldsymbol{u}_n||^2$. The coefficient of this term is chosen large but finite, allowing for a slight indentation of the border where it is pulled by the margin, as seen in experiments. The initial conditions for tension and myosin are as for the intact embryo, with the range of θ restricted accordingly.

c. Intact embryo with obstacle (main Fig. 5, Supplementary Video 7) In this case the embryo is modeled as in the intact case, except that the Stokes equation Eq. (30) is modified to include an additional friction term $\Lambda \boldsymbol{u}$ that is locally confined to a rectangular domain that covers the embryo fully in the lateral direction and is confined to a narrow window of twice the margin thickness, $2d = 0.24$ mm in the A-P direction, centered on the epiblast. The dimensionless friction coefficient $\Lambda = \mathcal{O}\left(10^3\right)$ is chosen sufficiently large to reduce tissue velocities by a factor of 10 in the frictional domain. For the case of the obstacle being placed across half the epiblast only, we modify this window accordingly. Using the same parameter values for simulation as elsewhere, we observe the phenotype that exhibits an ectopic contraction on the anterior side of the obstacle, i.e. the case in which tension does not propagate sufficiently far along the margin to suppress an ectopic contraction. By decreasing the ratio of tissue to margin viscosity $\mathcal{T}$ and the contractility amplitude to ζ by 20% each and leaving all other parameters unchanged, we obtain an alternative phenotype in which tension propagates far enough along the margin to suppress this contraction. In terms of our model, the reduction of $\mathcal{T}$ achieves the increased range of tension propagation, while maintaining a constant ratio $\zeta/\mathcal{T}$ ensures that the scales for velocities and contraction rates remain unaffected (c.f. Eq. (44)). This shows that our default parameters are marginal for this condition.

[6] M. Saadaoui, D. Rocancourt, J. Roussel, F. Corson and J. Gros. *A tensile ring drives tissue flows to shape the gastrulating amniote embryo.* Science **367**, 6476, 2020.

[24] N. Noll, M. Mani, I. Heemskerk, S. J. Streichan and B. I. Shraiman. *Active tension network model suggests an exotic mechanical state realized in epithelial tissues.* Nature physics **13**, 12, 2017.

[34] M. S. Alnaes, J. Blechta, J. Hake, A. Johansson, B. Kehlet, A. Logg, C. Richardson, J. Ring, M. E. Rognes and G. N. Wells. *The FEniCS Project Version 1.5.* Archive of Numerical Software 3, 2015.

[35] A. Logg, K.-A. Mardal, G. N. Wells et al. *Automated Solution of Differential Equations by the Finite Element Method.* Springer, 2012.

III. SUPPLEMENTARY TABLES

Parameter	Meaning	Value
α	Regulation inflexion	-1
β	Regulation sensitivity	16
ζ	Regulation amplitude	1/3
$\mathcal{D}$	Myosin diffusivity	7.5×10^{-4}
$\mathcal{P}$	Propagation number	2/3
δ	Margin thickness	0.208
λ	Walking kernel non-linearity	4
$\mathcal{E}$	Margin stiffness	1
$\mathcal{T}$	Tissue viscosity	0.77
L	Margin length	5.78 mm
d	Margin width	0.12 mm
$\sqrt{D\tau}$	Diffusion length	0.106 mm
τ	Regulation time scale	0.5 hours
$\dot{\epsilon}_0$	Maximal contraction rate	0.33 hours^{-1}
ν/E	Margin viscoelastic time scale	0.5 hours

Supplementary Table 1: Non-dimensional parameters used for numerical finite element simulations. Applicable to the model equations Eqs. (35a)-(35c) (top section) and a set of corresponding dimensional quantities (bottom section) that recapitulates experimental observations.

Mode	Meaning	Value	Coefficient
1	EP expansion	$\frac{1}{2}\left(1-\tanh\left(2(\sqrt{x^2+(y-d_s)^2}-u_e)/w_e\right)\right)$	0.27/8
2	A-P bias	$-\frac{y}{2}\left(1-\tanh\left(2(\sqrt{x^2+(y-d_s)^2}-u_e)/w_e\right)\right)$	0
3	EE expansion	$1-\frac{1}{2}\left(1-\tanh\left(2(\sqrt{x^2+(y-d_s)^2}-u_e)/w_e\right)\right)$	1.58/8
4	Outer boundary correction	$-\exp\left(-0.5\left(\sqrt{x^2+y^2}-e_r\right)^2/w_b^2\right)$	1.06/8
5	PS ingression	$-\exp\left(-0.5\left(\sqrt{x^2+(y-d_s)^2}-r_s\right)^2/w_s^2-0.5\left(\tan^{-1}\left(x/(y-d_s)\right)\right)^2/\theta_s^2\right)$	see caption

Supplementary Table 2: Modes of the tissue growth term γ, defined in accordance with the synthetic embryo [6]. The fifth mode is time dependent with amplitude $\frac{2.26}{2.9}\left(1+\tanh\frac{2\times(t-5.1)/2.0}{2}\right)$; as noted in Methods and Supplementary Methods II , it is omitted in simulations of posterior halves (Fig. 5) and with a full obstacle (Fig. 6). Additional parameter values taken from fit in[6]: $u_e = 1.0488$, $w_e = 0.3956$, $d_s = 0.106$, $e_r = 1.81$, $w_b = 0.19$, $r_s = 0.8694$, $w_s = 0.1196$ (all in mm), $\theta_s = 0.75$ radians.
