# Supplementary material for: Self-organized tissue mechanics underlie embryonic regulation
Source: Nature. 2024 Sep 11;633(8031):887–94. doi: 10.1038/s41586-024-07934-8 (PMC11424473; doi:10.1038/s41586-024-07934-8)
Supplement: Supplementary file 2 — Reporting Summary [file 41586_2024_7934_MOESM2_ESM.pdf]

nature portfolio

Corresponding author(s): Jérôme Gros & Francis Corson

Last updated by author(s): 13/06/2024

Reporting Summary

Nature Portfolio wishes to improve the reproducibility of the work that we publish. This form provides structure for consistency and transparency in reporting. For further information on Nature Portfolio policies, see our Editorial Policies and the Editorial Policy Checklist.

Statistics

For all statistical analyses, confirm that the following items are present in the figure legend, table legend, main text, or Methods section.

n/a	Confirmed	
☐	☒	The exact sample size (*n*) for each experimental group/condition, given as a discrete number and unit of measurement
☐	☒	A statement on whether measurements were taken from distinct samples or whether the same sample was measured repeatedly
☐	☒	The statistical test(s) used AND whether they are one- or two-sided<br>*Only common tests should be described solely by name; describe more complex techniques in the Methods section.*
☒	☐	A description of all covariates tested
☒	☐	A description of any assumptions or corrections, such as tests of normality and adjustment for multiple comparisons
☐	☒	A full description of the statistical parameters including central tendency (e.g. means) or other basic estimates (e.g. regression coefficient) AND variation (e.g. standard deviation) or associated estimates of uncertainty (e.g. confidence intervals)
☐	☒	For null hypothesis testing, the test statistic (e.g. *F*, *t*, *r*) with confidence intervals, effect sizes, degrees of freedom and *P* value noted<br>*Give P values as exact values whenever suitable.*
☒	☐	For Bayesian analysis, information on the choice of priors and Markov chain Monte Carlo settings
☒	☐	For hierarchical and complex designs, identification of the appropriate level for tests and full reporting of outcomes
☒	☐	Estimates of effect sizes (e.g. Cohen's *d*, Pearson's *r*), indicating how they were calculated

Our web collection on statistics for biologists contains articles on many of the points above.

Software and code

Policy information about availability of computer code

Data collection	Data was collected using a Zeiss microscope LSM 900 and LSM980 and its associated software ZEN.
Data analysis	Custom codes were used to analyze microscopy data.<br>See https://github.com/achamolly/mechanical_self_organisation/

For manuscripts utilizing custom algorithms or software that are central to the research but not yet described in published literature, software must be made available to editors and reviewers. We strongly encourage code deposition in a community repository (e.g. GitHub). See the Nature Portfolio guidelines for submitting code & software for further information.

Data

Policy information about availability of data

All manuscripts must include a data availability statement. This statement should provide the following information, where applicable:

- Accession codes, unique identifiers, or web links for publicly available datasets
- A description of any restrictions on data availability
- For clinical datasets or third party data, please ensure that the statement adheres to our policy

The datasets presented and analysed during the current study are available at: dx.doi.org/10.6084/m9.figshare.26004184

Research involving human participants, their data, or biological material

Policy information about studies with human participants or human data. See also policy information about sex, gender (identity/presentation), and sexual orientation and race, ethnicity and racism.

Reporting on sex and gender	N/A
Reporting on race, ethnicity, or other socially relevant groupings	N/A
Population characteristics	N/A
Recruitment	N/A
Ethics oversight	N/A

Note that full information on the approval of the study protocol must also be provided in the manuscript.

Field-specific reporting

Please select the one below that is the best fit for your research. If you are not sure, read the appropriate sections before making your selection.

☒ Life sciences ☐ Behavioural & social sciences ☐ Ecological, evolutionary & environmental sciences

For a reference copy of the document with all sections, see nature.com/documents/nr-reporting-summary-flat.pdf

Life sciences study design

All studies must disclose on these points even when the disclosure is negative.

Sample size	Sample size was not predetermined and results from the repetition of experiments, as permitted by the many steps involved in each individual experiment .
Data exclusions	No data was excluded from the analyses.
Replication	All experiments were replicated at least 6 times and up to 33 times.
Randomization	N/A. Transgenic embryos are used in this study and cannot be randomized. Transgenic embryos expressing the transgene were collected and used in experiments.
Blinding	Given the nature of experiments, for which perturbations are readily distinguishable from controls, (i.e. epiblast bisection, hair deposition, etc.) blinded analysis is not suited for this type of experiments.

Reporting for specific materials, systems and methods

We require information from authors about some types of materials, experimental systems and methods used in many studies. Here, indicate whether each material, system or method listed is relevant to your study. If you are not sure if a list item applies to your research, read the appropriate section before selecting a response.

Materials & experimental systems

n/a	Involved in the study	
☐	☒	Antibodies
☒	☐	Eukaryotic cell lines
☒	☐	Palaeontology and archaeology
☐	☒	Animals and other organisms
☒	☐	Clinical data
☒	☐	Dual use research of concern
☒	☐	Plants

Methods

n/a	Involved in the study	
☒	☐	ChIP-seq
☒	☐	Flow cytometry
☒	☐	MRI-based neuroimaging

Antibodies

Antibodies used	Antibody against Brachyury (Biotechne #AF2085, dilution 1/200) ; anti-DIG antibody (Biotechne #MAB7520, dilution 1/2000)

Validation	These antibodis have been validated "using direct EliSA and Western BLOT" as stated by the manufacturer statement Furthermore, the expression of Bra revealed in our study matches previously reported pattern and is congruent with RNA expression.

Animals and other research organisms

Policy information about studies involving animals; ARRIVE guidelines recommended for reporting animal research, and Sex and Gender in Research

Laboratory animals	Experiments in this study involve transgenic unincubated (0 day-old) quail embryos (coturnix japonica) carrying a membrane-bound eGFP reporter gene.
Wild animals	no wild animals were used in the study.
Reporting on sex	This information has not been collected.
Field-collected samples	No field collected samples were used in the study.
Ethics oversight	The generation and housing of transgenic qua il lines were approved by the Institut Pasteur Ethics committee Dha 210003 and by GMO Agreement #2432.

Note that full information on the approval of the study protocol must also be provided in the manuscript.

Plants

Seed stocks	N/A
Novel plant genotypes	N/A
Authentication	N/A
